# Covid-19 hotlines, helplines and call centers: a systematic review of characteristics, challenges and lessons learned

**DOI:** 10.1186/s12889-024-18702-8

**Published:** 2024-04-28

**Authors:** Maryam Eslami Jahromi, Haleh Ayatollahi, Ali Ebrazeh

**Affiliations:** 1 Department of Health Information Management, School of Health Management and Information Sciences, Iran University of Medical Sciences, Tehran, Iran; 2 Health Management and Economics Research Center, Health Management Research Institute, Iran University of Medical Sciences, No. 4, Rashid Yasemi St, Vali-Asr St, Tehran, 1996713883 Iran; 3 Department of Public Health, School of Public Health, Qom University of Medical Sciences, Qom, Iran

**Keywords:** Call centers, Covid-19, Hotlines

## Abstract

**Background:**

During the Covid-19 pandemic, a number of hotlines/helplines/call centers was implemented to provide remote services and support public health. The objective of this study was to investigate the characteristics, challenges and lessons learned of implementing Covid-19 hotlines/helplines/call centers during the pandemic.

**Methods:**

PubMed, Web of Science, Scopus, the Cochrane Library, IEEE Xplore, and ProQuest databases as well as Google Scholar were searched between 1st January 2020 and 31st December 2023 to retrieve relevant articles published in English. The quality and risk of bias of the studies were assessed using the Appraisal tool for Cross-Sectional Studies (AXIS), the Mixed Methods Appraisal Tool (MMAT), and Critical Appraisal Skills Programme (CASP) Checklist.

**Results:**

In total, 43 out of 1440 articles were included in this study. About half of the hotlines/helplines/call centers were launched in March 2020 (*n* = 19). Providing psychological support (*n* = 23), reliable information about Covid-19 (*n* = 10), healthcare advices about Covid-19 (*n* = 8), and triage (*n* = 7) were the most common purposes of implementing these services. The most common challenges included a lack of physical examination, unavailability of hotlines/helplines/call centers at the point of need, and delay in updating Covid-19 information. The most common lessons learned were employing qualified staff, providing proper training, and getting feedback from the callers and operators.

**Conclusion:**

According to the results, most of the Covid-19 hotlines/helplines/call centers were launched in the early months of the pandemic, and about half of them were active seven days a week. Most of the operators were mental health providers and clinicians. The findings show the importance of continuous psychological support during crises, particularly when adequate information about the situation is not available. The challenges experienced by the callers and operators as well as the lessons learned by the service providers also need to be considered for future crises to increase the effectiveness of similar services.

**Supplementary Information:**

The online version contains supplementary material available at 10.1186/s12889-024-18702-8.

## Background

The healthcare system must be ready to respond to a wide range of emergencies and disasters that may threaten public health [1]. In fact, strong healthcare preparedness is essential for effective disaster response [2]. Natural hazards, including hurricanes, earthquakes, tornadoes, fires, and floods, may pose significant and varied risks across the countries. In addition, human and animal infectious diseases, including those previously undiscovered, may present considerable risks to the communities. Technological and accidental hazards, such as dam failures or chemical substance spills or releases, may also have the potentials to cause extensive fatalities. These issues show the importance of a flexible healthcare system, and federal, state, and local governmental agencies are responsible for planning, training, and exercising for emergencies and disasters [3].

During an emergency, especially those that are primarily health-focused, the public will often and inevitably ask the public health communities for guidance and assistance [4]. One of the main solutions to respond to the public health queries is implementing hotline services [5]. These services have been used for more than 60 years and have helped people to receive accurate and timely health information to make informed decisions about their conditions [6]. Health call centers or hotlines have the potential to be particularly impactful in strengthening health systems in low- and middle-income countries, as they allow users to call and receive health advice over the phone. Offering access to health information over the phone mitigates some challenges, such as transportation costs, cost of in-person visits, and healthcare professional shortages. Additionally, the anonymous nature of a hotline may alleviate stigma and nervousness that could prevent a client from discussing a sensitive health topic with a local healthcare provider [7]. Health hotlines/helplines/call centers differ on a variety of characteristics, including operating hours, purposes, or the health topics that they cover, operators’ characteristics, and target groups. While call centers in high- income countries tend to host more all-purpose hotlines, the hotlines in low-income countries are typically designed to cover specific health topics. In sub-Saharan Africa, for instance, individual hotlines have traditionally focused on one specific content area such as maternal health education, antiretroviral adherence, management of non-communicable diseases, or triaging post-operative adverse events [8].

Apart from many public health crises which have been experienced by different countries, in the 21st century, three respiratory pandemic diseases, namely Severe Acute Respiratory Syndrome (SARS), Middle East Respiratory Syndrome (MERS), and Covid-19, have impacted human life [9]. The last one was first reported in Wuhan, China in late December 2019, and on 11th March 2020, the World Health Organization (WHO) declared Covid-19 as a global pandemic [10]. Statistics indicate that more than 769 million people have been infected with Covid-19 across the globe, and more than six million deaths have been reported to the WHO throughout the world [11].

During the outbreak of the disease, different countries implemented various strategies including patient quarantine, controlling individuals’ movement, closure of schools and restaurants, restrictions on international travel, use of masks, social distancing, as well as providing online self-assessment tools and helplines to control the spread of the disease [12, 13]. The implementation of hotlines/helplines was in line with one of the most important WHO strategies; namely providing accurate information to the public, because information plays a crucial role in disease control and protection of individuals against the disease. Although social networks and messaging apps play a crucial role in disseminating information, sometimes they can be effective in spreading false information that may endanger public health [14]. Studies have shown that during the outbreak of a new disease, phone lines are an important and accessible resource for providing information to reduce public panic [5, 15–17].

During the Covid-19 pandemic, the World Health Organization’s Regional Office for Europe developed guidelines for the establishment and management of Covid-19 hotlines and call centers [18]. Examples of the launched phone lines and call centers in different countries include the 1339 hotline in South Korea [19], a special hotline in China [5], Covid-19 call centers in the United States [20], Germany [21], Bolivia [22], the 937 call center in Saudi Arabia [23], and the 4030 call center in Iran [24]. The use of phone lines could reduce public anxiety during the pandemic and provided accurate information while answering people’s questions. In addition to incoming calls, some hotlines operators were calling patients for the screening process and identifying positive Covid-19 cases [24–26]. in addition, data obtained from people’s calls could help others. Daily collection and weekly sharing of information from callers could reveal emerging trends in worries and attitudes and help policymakers to take effective actions to be more responsive [5]. It should be noted that many of health strategies, in addition to their advantages, may have unintended consequences, which makes it necessary to conduct periodic and comprehensive evaluations [27, 28].

Previously, a number of studies have been conducted to describe the use of call centers and phone lines, especially during the Covid-19 pandemic [21, 22, 29, 30]. For instance, Nina-Mollinedo et al. reported beneficial findings regarding the tracking of suspected Covid-19 cases at the national level through early diagnosis by the Covid-19 call center of the Ministry of Health and Sports in Bolivia [22]. In another study, Vonderlin et al. evaluated the psychological hotline during the first wave of the Covid-19 pandemic. The results indicated that delivering psychological services via phone was feasible in pandemic conditions and played an important role in overcoming individuals’ psychological stress [21]. In Cheng et al.’s study, the results showed that the assessment of a phone line for Covid-19 primary care in Oregon state met the public’s need for information and access to primary care. The findings demonstrated that further investigations on the factors influencing the success or failure of this strategy are necessary [31].

According to the best of our knowledge, no systematic review study has been conducted or officially published on the implementation of hotlines/helplines/call centers during the Covid-19 pandemic. Therefore, the aim of the present study was to systematically review the characteristics, challenges, and lessons learned from implementing these services during the Covid-19 pandemic. In this study, hotlines/helplines/call centers that were set up specifically for Covid-19 and related issues during the pandemic were investigated. The results of this study can contribute to improve the theoretical knowledge on the implementation of these services during the pandemics.

## Methods

This systematic review was completed in 2024. Prior to conducting the research, the ethics approval was obtained from the National Ethics Committee of Biomedical Research (IR.IUMS.REC.1401.332). This review study was conducted in accordance with the PRISMA 2020 statement: an updated guideline for reporting systematic reviews [32].

### Protocol registration

The study protocol was registered in INPLASY, an international platform for registration of systematic review and meta-analysis protocols (Registration number: INPLASY202420052, DOI number: 10.37766/inplasy2024.2.0052).

### Identifying the research question

In a review study, the starting point is to identify the research question to be able to develop the search strategies. The initial literature search suggested that the information about the implementation of Covid-19 hotlines, helplines and call centers were fragmented; therefore, we generated a research question as follows:

What were the characteristics of Covid-19 hotlines/helplines/call centers?

What were the challenges of implementing Covid-19 hotlines/helplines/call centers?

What were the lessons learned of implementing Covid-19 hotlines/helplines/call centers?

### Eligibility criteria

The timeframe of the study was between 1st January 2020 and 31st December 2023. To select the most relevant studies, some inclusion and exclusion criteria were set. Then, all research papers, reviews, conference papers, theses, and dissertations which were related to the Covid-19 hotlines, helplines and call centers, and their full-texts were available were included in the study. In addition, we decided to include only articles which were published in English. In fact, choosing English language articles is common in conducting reviews and may have minimal effect on overall conclusions [33]. Moreover, a limited number of papers were in non-English languages, and we had limited resources to be able to translate these papers. Furthermore, protocols, reports, letters to the editor, and studies in which hotlines, helplines or call centers were used in fields other than Covid-19 were excluded from the study.

### Information sources

Six databases, namely, PubMed, Web of Science, Scopus, the Cochrane Library, IEEE Xplore, and ProQuest databases, as well as Google Scholar were systematically searched from 1st January 2020 until 31st December 2023. In addition, the Open Grey database was searched to find the grey literature. The search process was also carried out by reference and citation checking. If the full text of an article was not available, the corresponding author was contacted.

### Search strategy

To develop a search strategy, MeSH (Medical Subjects Headings) Terms such as Covid-19, coronavirus, severe acute respiratory syndrome, SARS-CoV-2, hotline, and call center, as well as key terms such as Corona, 2019-nCoV, Covid, helpline, crisis line, and emergency line were identified, and combined using “AND” and “OR” operators. The list of databases and used search strategies are presented in Appendix I.

The selection process was performed in accordance with the Preferred Reporting Items for Systematic Reviews and Meta-analyses (PRISMA) 2020 flow diagram [32]. After retrieving relevant articles, the EndNote software (Version X8) was used, and duplicates were removed. The initial search was conducted by (MEJ) and the screening processes were completed by both authors (MEJ and HA). The authors independently screened the title, abstract, and full text of all eligible articles, and any disagreements were resolved through discussion between the two researchers and reaching a consensus or discussing the issue with the third author (AE). Both authors had a related background to the topic of the study.

### Data collection process

Data were extracted using a data extraction form which consisted of the name(s) of the author(s), year of publication, country, research objective, research methods, name of the hotline, target users, activation period, hotline access time, purpose of implementing the hotline, reasons for call, key findings, challenges, and lessons learned. Other data, such as the methods for service promotion, number of calls, call agents’ (operators’) profession, service provider, and quality of services were also extracted. The first author (MEJ) initially collected the data, and the reports were reviewed independently by authors (HA) and (AE). In case of disagreement, the researchers discussed the issue and resolved it by reaching a consensus.

### Data items

In this study, the characteristics of Covid-19 hotlines and call centers, challenges and lessons learned were the main data items that were examined and compared in different studies.

### Study quality and risk of bias assessment

Quality assessment was performed by two researchers (MEJ and HA) independently and any disagreement between the researchers was resolved by discussion. As different research methodologies were used in the reviewed articles, the Appraisal tool for Cross-Sectional Studies (AXIS) was used to assess the risk of bias, quality of design, and quality of reporting in quantitative studies [34]. The tool had 20 questions, and each question had three responses (yes (1), no and don’t know (0)). Each individual study received a score between zero and 20. Based on these scores, the individual studies were categorized into three groups: Good (> 15), fair (10–15) & poor (< 10). As mixed-methods methodology was used in one study, its quality was assessed using the Mixed Methods Appraisal Tool (MMAT) [35]. It consists of five questions with “yes”, “no” and “can’t tell” as the response options. Using this tool, the quality of an article can be assessed as zero, 25%, 50%, 75% and 100% (zero (no criterion met), 25%, 50%, 75% and 100% (all criteria met)). Indeed, a higher score indicates higher quality. As some of the included studies used qualitative designs, the Critical Appraisal Skills Programme (CASP) Checklist were used to assess the quality and risk of bias for these papers [34]. It consists of 10 questions, with “yes,” “no,” or “can’t tell” as the answer options. The calculated scores showed the quality of each study as high (7–10), medium (4–6), or low (1–3).

### Synthesis methods

As different qualitative, quantitative, and mixed methods studies were included in the current research, we were not able to conduct a meta-analysis. Therefore, to report the results, the characteristics of Covid-19 hotlines, helplines, and call centers challenges, and learned lessons were described. To summarize data, tables were developed based on the data extraction form, and finally, the results were synthesized narratively.

## Results

### Study selection

**Fig. 1:**
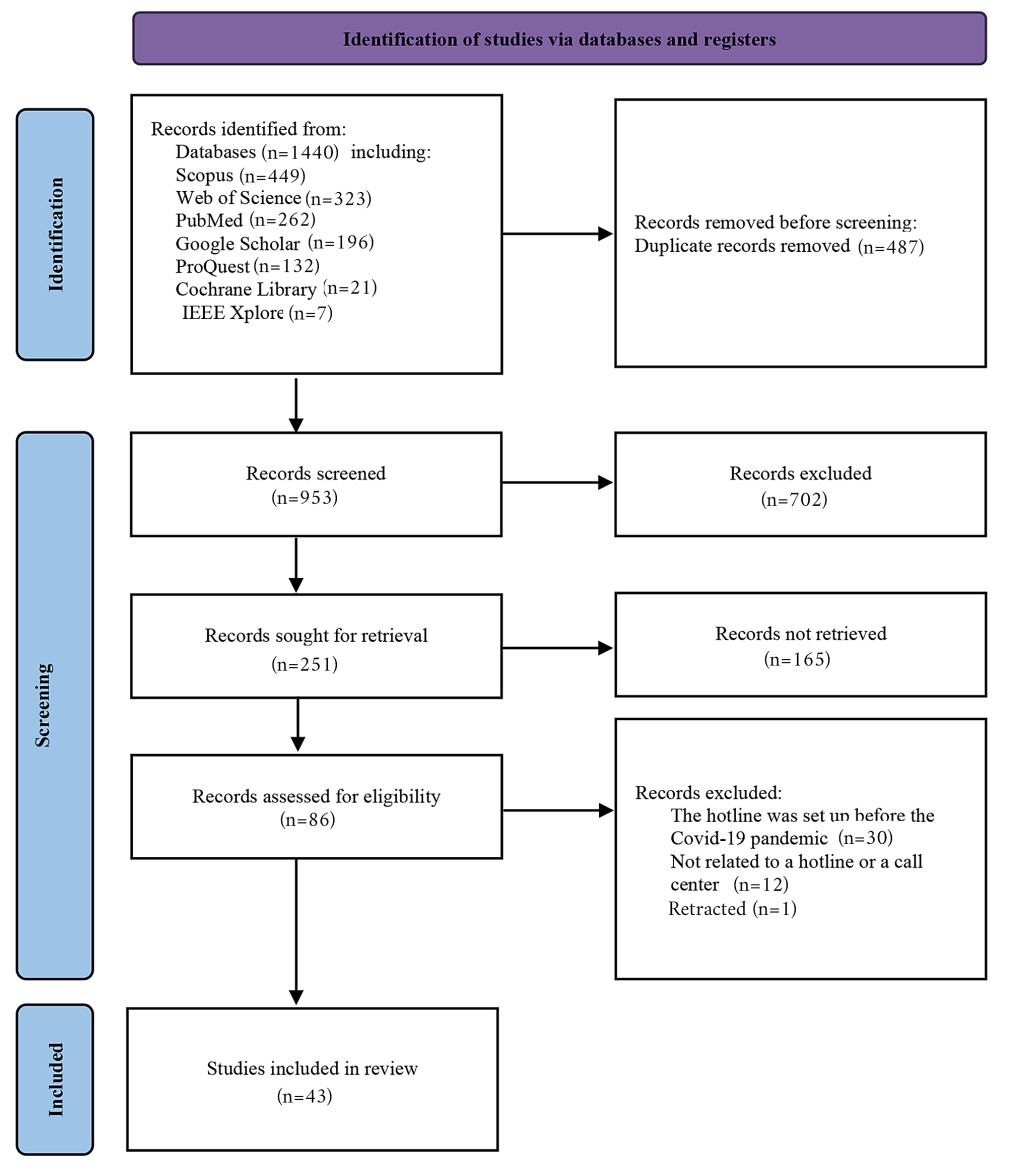
Article selection process based on the Preferred Reporting Items for Systematic Reviews and Meta-Analyses (PRISMA)

In this study, 1440 articles were retrieved through searching six databases and Google Scholar. Initially, all articles were entered into the EndNote software (Version X8), and duplicates (*n* = 487) were removed. Then, the relevancy of the remaining articles to the study objective was examined based on their titles and abstracts, and 867 articles were excluded. The full texts of the remaining articles (*n* = 251) were searched, and 165 articles were not retrieved. The full texts of the remaining articles (*n* = 86) were reviewed, and 43 articles were removed as their full text did not meet the inclusion criteria. Finally, 43 articles were selected to be included in the current study (Fig. 1).

### Study characteristics

As Table 1 shows 18 Studies were undertaken in the Asia (India [36–43], China [30, 44–47], Qatar [48, 49], Bangladesh [50], Indonesia [51], Nepal [52]), 11 in the North America (United States [17, 20, 29, 31, 53–58], Dominican Republic [59]), 10 in the Europe (Austria [60], France [61], United Kingdom (UK) [62, 63], Slovenia [64], German [65], Ireland [66], Poland [16], Serbia [67], Spain [68]), 3 in Africa (Tunisia [69], Egypt [70], Uganda [71]), and 1 in South America (Bolivia [22]). Among them, 11 studies were published in 2020, 11 in 2021, 9 in 2022, and 12 in 2023, and a summary of them is presented in Table 1.

**Table 1:** Summary of the selected articles

No	Author (Year)	Country	Objective	Research methods	Name of the hotline/Target users	Activation period/ Access time	Purpose of implementing the hotline	Reasons for call	Key findings
1	Bric and Raile, 2020 [60]	Austria	Describing the experience of setting up an innovative psychotherapeutic helpline during the Covid-19 crisis	Quantitative	Psychotherapeutic helpline / Public	March 21st until May 29th, 2020 / Seven days a week, from 10 a.m. to 4 p.m.	Psychotherapy via telephone in terms of insecurity and fear during the Covid-19 crisis	Psychological issues, family problems, sleep problems, physical discomfort, financial and work difficulties	The symptoms most callers described were panic attacks. The clients accepted the offer very positively and were also satisfied with the respective care. Professionals also praised the initiative, emphasizing that they enjoy their work.
2	Carson et al., 2020 [53]	USA	Assessing medical students’ comfort level with several hotline tasks before and after their experience as a hotline volunteer	Quantitative	Covid-19 hotline/ Underserved rural populations of northern Nevada	March 2020 until now	Addressing residents’ questions, providing information from reliable resources, reducing the burden on the local health care system, and triaging patients	Covid-19 queries	Participation in the multicounty Covid-19 hotline improved students’ (who worked on the hotline) comfort levels in all areas, with significant improvement in answering questions about SARS-CoV-2, screening and triaging patients, conducting audio-only exams, and addressing financial barriers to care.
3	Geoffroy et al., 2020 [61]	France	Presenting the methods for implementing Covid-Psy hotline support system and characterize first calls and reasons for the call	Mixed-methods	Covid-Psy hotline/ Hospital workers	March 2020 until now/ 24/7	A psychological support system in order to prevent or early intervene in case of mental health problems for all hospital workers during the Covid-19 outbreak	Psychological issues, Covid-19 queries, sleep problems	Mostly women (86%) called the hotline. Anxiety symptoms (49%) were the first cause for hospital workers to call the hotline. This psychological support system could be used by all hospital professions that all appeared psychologically affected.
4	Joshi et al., 2020 [36]	India	Exploring experiences and challenges faced by counselors who are working with a national-level Covid-19 telephone counseling helpline in India	Qualitative	iCALL Covid-19 helpline/ Public	April 2020 until now	Assisting individuals and communities with emotional and practical support	Psychological issues	The nature of concerns presented by the callers were often a mix of psychological, relational and practical issues.
5	Kristal et al., 2020 [17]	USA	Describing New York’s hotline and public health in the rapidly changing Covid-19 pandemic	Qualitative	NYC H + H Covid-19 hotline/ Public	March 2020 until now	Assessing Covid-19 related concerns and providing clinical and informational guidance to New Yorkers.	Psychological issues, Covid-19 queries	The hotline provided New Yorkers with an up-to-date source for Covid-19 clinical and informational guidance. Hotline clinicians were able to reassure the callers with no or mild symptoms, and help those who truly needed emergency medical services.
6	Kumar et al., 2020 [62]	UK	Describing the setting up a telephone support hotline for Covid-19 and respiratory enquiries at a large NHS trust in response to the Covid-19 pandemic	Quantitative	Respiratory specialist hotline / All GPs in the Northwest London, health and care partnerships, GP practice nurses and community pharmacists	April 2020 until now/ Seven days a week, from 9 a.m. to 6 p.m.	Respiratory specialist advice, second opinions and practical advice on managing new and challenging clinical situations both in relation to Covid-19 and in the context of other general, non-Covid-19 respiratory queries	Covid-19 queries	Feedback from the GPs who have used the service has been encouraging. Satisfaction with the service received a mean score of 9.3/10. GPs felt that the hotline was effective in changing clinical management (8.5/10), providing ease of access to the specialist advice (8.8/10), improving confidence in managing respiratory manifestations of Covid-19 (8.7/10), ensuring coordination of care (8.6/10), and helping GPs to feel supported (8.8/10).
7	Margolius et al., 2020 [54]	USA	Examining the effectiveness of the first five weeks’ of a 24/7 physician-staffed Covid-19 hotline	Quantitative	Covid-19 hotline/ Public	March 2020 until now/ 24/7	Assessing, advising and treating individuals who called with symptoms that could be Covid-19 related	Covid-19 queries	Common caller concerns included cough (22.3%), fever (4.7%), and shortness of breath (6.1%). Most callers (79%) were advised to self-isolate at home, and only 3% were advised to immediately seek care via emergency department. Telephone hotline services conserved scarce resources and provided effective, equitable care during a pandemic without compromising patient safety.
8	Matthewson et al., 2020 [63]	UK	Describing the implementation and analysis of a psychology-led Covid-19 telephone support line in an NHS occupational health setting	Mixed-methods	Support line/ Staff members employed by a large acute NHS trust	March 2020/ until now/ Monday to Friday, from 09:00 a.m. until 17:00 p.m.	Providing individuals with emotional support	Psychological issues, Covid-19 queries	The service provided responsive communication and supported the staff who might be emotionally affected by Covid-19. The majority of calls were requests for practical information in the form of clarification of guidance (68%). Most requests for information were on what to do when symptomatic (39%).
9	Pelicon et al., 2020 [64]	Slovenia	Examining the impact of a centralized novel coronavirus telephone helpline on managing the Covid-19 health crisis by shaping and monitoring the public’s response	Quantitative	National coronavirus helpline/ Public	9th March until 12 th June 2020/ Seven days a week, from 8 a.m. to 8 p.m.	Creating a one-stop-shop for citizens with questions on the Covid-19 pandemic	Covid-19 queries Social problems, movement restriction	The helpline represented one of the first points of contact for the shocked and unprepared citizens seeking reassurance and help. Content of the calls changed in accordance with the progression of the epidemic and governmental restrictions. Initially, a considerable pro-portion of callers sought medical advice with questions about Covid-19 signs and symptoms, viral transmission, and preventive measures in March. Towards the end of March 2020, the focus of the queries shifted to mitigation measures such as quarantine and restrictions on public movement.
10	Ravindran et al., 2020 [37]	India	Describing the preliminary experience in providing psychosocial support amid the Covid-19 pandemic from a tertiary care centre in India	Quantitative	Psychosocial Support and Mental Health Services (PSSHMS) helpline/ Public	April 2020/24/7	Providing psychosocial support and mental health services and addressing the mental health related concerns of the citizens during the Covid-19 pandemic lockdown	Psychological issues, movement restriction	Over 90% of callers were satisfied with the provision of the service. Callers reported that the mental health professionals were able to address their psychological distress and help with linking to local resources, thereby addressing their concerns. Most of them reported that they would call back the helpline in crisis.
11	Shao et al., 2020 [44]	China	Summarizing Taizhou’s Covid-19 prevention and control experience with telemedicine features, with a view to providing reference for the control of the epidemic at home and abroad	Quantitative	Covid-19 prevention and treatment special line / Public	January 2020 until now/ 24/7	Giving the public the corresponding professional consultation, follow-up, intervention, and guidance online. Completing prehospital screening and early detection and treatment	Covid-19 queries	Covid-19 prevention and treatment special line was created for pre-hospital screening, suppressing social panic, and clinical support.
12	Abdelghaffar et al., 2021 [69]	Tunisia	Describing the design, implementation and activities of psychological support unit (PSU) with a free helpline	Qualitative	Psychological support unit (PSU) helpline/ Patients, their families and health workers	April 2020 until now	Facilitating access to healthcare services including mental health services during the lockdown particularly for Covid-19 patients.	Psychological issues	There was widespread positive feedback from the patients and professionals concerning the psychological support unit (PSU) and its services.
13	Abdullah et al., 2021 [29]	USA	Describing the development, implementation and outcomes of a Covid-19 anxiety hotline	Mixed methods	Covid-19 anxiety hotline/ Public	April 2020 until now/ 24/7	Addressing the community’s mental health support provoked by the coronavirus pandemic	Psychological issues, sleep problems	The Covid-19 mental health hotline appears to have successfully targeted individuals in both the healthcare settings and in the general population. The number and variety of calls suggests that the service was utilized well by the target populations. Anxiety and sleep disturbances were the most prevalent symptoms reported by the callers (72.2%).
14	Cheng et al., 2021 [31]	USA	Describing the launch of a statewide Covid-19 primary care hotline and telemedicine Service	Mixed-methods	Covid-19 connected care center hotline/ Public	March 2020 until now/ Seven days a week, from 8 a.m. to 8 p.m.	Addressing patients’ concerns about the Covid-19 pandemic	Covid-19 queries	Most of the patients (86%) reported that their questions were answered during the call, 90% would recommend this service, and 70% reported a reduction in their stress levels about Coronavirus. In qualitative interviews, patients reported that their questions were answered, waiting times were short, nurses spent time as needed, and appropriate follow-up was arranged.
15	Cher et al., 2021 [55]	USA	Describing the development and implementation of a Covid-19 patient triage hotline in a single health care system	Quantitative	Covid-19 triage hotline/ Patients	March 2020 until now/ Daily, from 6 a.m. to 12 a.m.	Triaging inbound patient calls related to Covid-19	Covid-19 queries	Cough (34%) was identified as the most common symptom. The Michigan medicine Covid-19 hotline effectively triaged patients seeking advice and care during the Covid-19 pandemic while facilitating characterization of local disease burden.
16	Du et al., 2021 [30]	China	Analysis of characteristic of callers to a psychological hotline at the early stage of Covid-19 in China	Mixed-methods	Psychological assistance hotline/ Public	February 2020 until now/ 24/7	Providing psychological services to the people in time to prevent their psychological problems from further deteriorating and appropriately alleviate their panic.	Psychological issues, sleep problems, Covid-19 queries	A total of 95.0% agreed that the hotline helped them. The public reported the persistent effectiveness of the hotline, which indicated that the hotline could be a powerful way to provide help during the pandemic.
17	Hazarika et al., 2021 [38]	India	Evaluating the sociodemographic profile of the distressed callers, their psychosocial concerns, the interventions provided by the service provider, and whether the service users were satisfied with the interventions or not	Mixed-methods	Covid-19 hotline/ Public	April 2020 until now/ from 9 a.m. until 9 p.m. (Active for 12 h per day)	Addressing the psychological issues	Psychological issues, Covid-19 queries, family problems, financial and work difficulties	Overall, most of the callers (49.8%) were satisfied and appreciated (42.3%) the tele-counselling services. The majority of the callers were male (79.1%). Most of the callers were between 19–35 years old (66.5%), married (52.5%), and graduates (31%). The majority of the callers had symptoms of anxiety (46%). The commonest intervention provided to the callers was supportive therapy (77.8%).
18	Iqbal et al., 2021 [50]	Bangladesh	Describing the Covid-19-related issues raised by callers on tele‐counseling helpline in Bangladesh during lockdown	Mixed-methods	Moner Jotno Mobile E/ helpline/ Public	March 2020 until now/ from 8 am to 12 am every day	Providing mental health service due to the Covid-19 lockdown.	Psychological issues, Covid-19 queries, family problems, sleep problems, financial and work difficulties	Most of the callers to this helpline were male (71%) and rural/subrural residents (68%). The vast majority (80%) of callers displayed anxiety and sleeplessness related to the lockdown.
19	Jang et al., 2021 [20]	USA	Reporting the experiences in creating the radiology call center and outpatient Covid-19 imaging sites during the Covid-19 healthcare crisis	Quantitative	Radiology Covid-19 call center/ Patients and health care providers	March 2020 until now/ 7 days a week (7am-7pm) until May 31, with hours reduced to Monday to Saturday (8am-5pm weekdays and 8am-3pm Saturday) on June 1. Beginning August 22, Saturday hours were also removed.	Answering all radiology questions related to Covid-19 and help with scheduling examinations	Covid-19 queries	This project provided efficient radiology operations during an emergency situation by providing a single reliable point of contact and a source of truth for all facets of radiology. Most common reasons for calling were related to scheduling (92%) and radiology operations (6%).
20	Meaden et al., 2021 [56]	USA	Describing the operation of the coronavirus hotline at the New Jersey Poison Information and Education System (NJPIES) through October 2020	Mixed-methods	New Jersey Coronavirus Hotline/ Public	January 2020 until now/ 24/7	Answering questions regarding the Coronavirus	Covid-19 queries	Public and healthcare providers made up 90% and 5.5% of callers. Most calls (68.7%) were regarding testing for Covid-19 and for general questions/symptoms. As cases of Covid-19 began to surge throughout the state, the call volume to the hotline dramatically increased. The New Jersey’s coronavirus hotline could serve as a model across the United States and around the world in operationalizing an emergency public health call center.
21	Wahl et al., 2021 [65]	German	Reporting results from a geriatric psychiatry helpline regarding the needs of the older adults, their reported changes, and the psychological impact during the initial stages of the health crisis	Mixed-methods	Covid-19 helpline/ Older adults	April 2020 until now/ Daily services from 8 a.m. until 5 p.m.	Offering psychosocial support and assessing the mental health impact of the health crisis among older adults	Psychological issues, Covid-19 queries	Helpline was able to rapidly respond to the specialized psychosocial needs during the crisis. Most reported reasons for calling the helpline were queries about coronavirus (60.7%). Most callers were women (85.5%) with an average age of 74.69, single, and retired. In total, 69% of callers reported new or an increase in psychiatric symptoms, with anxiety and depressive symptoms being the most common ones.
22	Zabrzygraj and Świtaj, 2021 [16]	Poland	Analyzing and summarizing the operation of the Mokotow helpline during the first wave of the Covid-19 pandemic	Mixed-methods	Mokotow helpline/Public	March 26th until July 31st , 2020/ Monday to Friday, from 8:00 a.m. to 8:00 p.m.	Supporting individuals by providing psychological help and psychoeducation or providing information on access to the appropriate health services	Psychological issues, Covid-19 queries	The Mokotow helpline was proved to be useful in providing support to the general public in the face of an unprecedented epidemiological crisis. The most common reasons for contacting the helpline were seeking advice and support for symptoms of mental disorders (21.4%). Emergency support was given to 70.6% of callers and regular short-term support was provided to 29.4%. Most of the interventions consisted of psychological support (73.8%). The helpline was contacted twice as often by women (63.7%) as men (32.8%).
23	Alfatih and Rachmawati, 2022 [51]	Indonesia	Analysing Covid-19 information services provided by the government, through the Covid-19 hotline	Mixed methods	Covid-19 hotline/Public	March 2020 until now/ 24/7	Providing Covid-19 information services	Covid-19 queries	The Covid-19 hotline had a positive impact in supporting the government’s efforts to suppress the spread of Covid-19. The hotline was able to provide the community with the information needs. The highest number of incoming calls for the hotline was in March 2020, with a total of 868 calls. 52% of callers used the hotline to obtain information related to Covid-19. The most dominant category of information asked by the public was health services (27%).
24	Egić, 2022 [67]	Serbia	Analyzing the structure of the questions asked on-call GPs in the Call center, formed in the city Town Hall	Quantitative	Covid-19 call center / Public	March 14th 2020 until May 04th 2022, Workday, from 08 h to 22 h, and on Saturdays from 08 h to 15 h.	Providing citizens with valid information on Covid-19 and other health issues	Psychological issues, Covid-19 queries, Financial and work difficulties, Social problems, Movement restriction	In the early phase of the pandemic, on-call GPs helped citizens solve different problems, even non-health ones. Healthcare workers and associates in primary healthcare, who worked in the call center, remained the frontline contact between patients and the health system in the early days of the pandemic. The highest percentage of questions were about fever and other symptoms that might be connected to Covid-19 (21.8%).
25	Khan et al., 2022 [39]	India	Understanding the current problems and perceptions about the Covid-19 pandemic within the public with analyzing the questions asked via the helpline	Quantitative	Covid-19 helpline/ Public	May 14th until May 16th 2021/ From 1–5 PM	Teleconsultations to Covid-19 patients	Vaccination, Covid-19 queries	There was equal distribution of calls from urban as well as rural areas. Urban callers were more interested in vaccination while rural inquiries related more to treatment and follow up of an active disease. Many had suffered despite being vaccinated. Among the sufferers 90% of people had undergone home based care. Active cases were mostly from rural areas.
26	Monreal-Bartolomé et al., 2022 [68]	Spain	Analysing the calls received during the Covid-19 lockdown by the mental health crisis helpline, studying the reasons for the calls, and describing the population segments that used it	Quantitative	Mental health crisis helpline (MHCH)/ Public	March 23rd until May 29th 2020/ Monday to Friday, from 8 a.m. to 8 p.m.	Psychological care of the population during the self-isolation and quarantine.	Psychological issues, Family problems	Women used the service almost three times more than men (73.91%). The majority of the calls were made by adults (77.9%), compared to the older people (19.1%) and the young people/children (3%). Anxiety symptomatology was the main concern of callers (38%). Significantly more men called for anxiety symptoms (60.8% vs. 49.5%) versus more women calling regarding a chronic physical illness (3.5% vs. 0.7%)
27	Nina-Mollinedo et al., 2022 [22]	Bolivia	Evaluating whether teleconsultation is helpful as an instrument of mediated care in the monitoring and follow-up of individuals with high suspicion of Covid-19 through early detection by the Covid-19 call center of the Ministry of Health and Sports, Bolivia	Quantitative	Covid-19 call center/ Bolivians	March 2020 until now	Monitoring and following-up through teleconsultation	Covid-19 queries	Teleconsultation as a tool for monitoring and following-up patients with high suspicion of Covid-19 was helpful. The ministry of health and sports reinforced the epidemiological surveillance system as a passive search tool for possible suspected cases at the national level and decongesting other services in charge of this task through the Covid-19 call center. The age range with the highest demand for the service was 30–44 years,50.6% were male, and 49.4% were female.
28	Ouyang et al., 2022 [45]	China	Analyzing the chief complaints of three psychological crisis hotlines during the Covid-19 pandemic in Jiangsu, China, and summarizing the psychological characteristics of the public during the different stages of Covid-19	Mixed-methods	Psychological crisis hotline/ Public	February 2020 until now/ 24/7	Providing professional psychological crisis intervention services to prevent psychological stress due to the epidemic	Psychological issues	The proportion of male callers (56.03%) was slightly higher than that of female callers during the Covid-19 outbreak period. Hotline callers were mainly young and middle-aged people (aged 19–45 years). Anxiety was the main complaint of the callers (64.01%). The monthly main complaints showed a fluctuating trend, and each main complaint peaked at different stages.
29	Peng et al., 2022 [46]	China	Exploring the psychological and emotional responses during different stages of the Covid-19 pandemic based on a survey of a mental health hotline	Mixed-methods	Mental health hotline/ Guangdong’s inhabitants	January until mid-August 2020	Providing emergency psychological assistance to alleviate the discomfort of the epidemic	Psychological issues, family problems, sleep problems, Covid-19 queries	The main problems of the callers seeking psychological assistance during the epidemic included comorbidities, anxiety, and sleep disorders. Different periods showed different main problems. Fear, worry, anxiety, and comorbidity accounted for the largest proportion in the early and middle stages of the epidemic. Self-harm or suicide were the most common in the middle of the epidemic. Family relationship problems were reported by most callers in the later stage of the epidemic.
30	Singh et al., 2022 [52]	Nepal	Describing the process of creating the hotline and preliminary findings from 10 months of free Covid-19 hotline phone service across Nepal	Quantitative	Covid-19 hotline/ Public	May 2021 until now/ 15.5 h a day (6 a.m. to 9:30 p.m.)	Teleconsultation services for those infected with Covid-19	Covid-19 queries	The hotline service was successful based on the number of calls answered and the utilization of the phone service widely during the peak of the pandemic. On average, there were 75 calls each day in the months of May and June 2021. The majority of the callers were male (58.6%), and 44% of the callers inquired about the clinical manifestations of Covid-19. Patients had a very positive response regarding this service.
31	Sosa Lovera et al., 2022 [59]	Dominican Republic	Describing the development, implementation and evaluation of Covid-19 helpline service in psychological first aid	Quantitative	UASD Covid-19 helpline University of Santo Domingo (UASD)/ Public	March to July 2020/ Monday to Wednesday, from 7 a.m. to 11 p.m.	Psychological first aid	Psychological issues, Covid-19 queries, family problems	More than 80% of the users felt that psychologists paid a lot of attention and interest. 81% of the respondents considered the given recommendations as useful, and 79% noted that their symptoms improved after the telephone intervention. Most people (96%) indicated they would call the helpline again if they felt emotionally unwell, and 97% said they would recommend it to other people.
32	Abdelbaky et al., 2023 [70]	Egypt	Evaluating the effectiveness of remote hotline emergency triage services during Covid-19 crisis in Upper Egypt	Quantitative	Hotline emergency triage/ Covid-19 infected patients	Not reported/ 24/7	Remotely monitoring patients’ vital signs and assessing their manifestations	Covid-19 queries	Using remote hotline emergency triage services was effective during Covid-19 crisis in Upper Egypt. About 75.1% of the study sample had satisfactory awareness and 50.1% had positive attitude towards using online emergency triage services.
33	Alabdulla et al., 2023 [48]	Qatar	Investigating the sociodemographic and clinical features of callers classed by the Qatar Helpline as moderate to high priority based on the risk of self-harm or suicide during the Covid-19 pandemic.	Quantitative	National Mental Health Helpline (NMHH)/ Public	April 2020/ six days a week	Addressing mental health and well-being issues for the Qatari community during the Covid-19 pandemic	Psychological issues	The majority of calls were made by patients (77.7%), followed by carers (20.1%). patients demonstrated predominantly positive feedback and 90% of callers stated that they would recommend the service to a friend or a relative. The most common symptoms in the patients included suicidal thoughts (73.1%), depressed mood (65.3%), disturbed sleep (58.6%) and anxiety (43.4%) and the most common psychiatric disorders were depression (30.5%), generalised anxiety (11.8%) and mixed anxiety and depressive disorders (11.6%).
34	Arafa et al., 2023 [49]	Qatar	Describing the initiation, feasibility, organization and effectiveness of a large-scale physician-staffed hotline to providing care and disseminating information during the first five months of the Covid-19 lockdown in Qatar	Quantitative	Urgent Consultation Center (UCC) hotline/ Public	April 2020/ Working Hours Initially, were 7am-10 pm, 7 days week, changed to 6 days week	To triage, consult, identify diseases, and treat /or refer according to the condition’s urgency	Repeat and new prescriptions for internal medicine geriatrics	Comprehensive and hospital-integrated hotline was feasible and can be employed in innovative ways to conserve medical resources, maintain continuity of care, and serve patients requiring urgent care during a pandemic. In this study 5% of calls were true emergencies which were advised to go to the emergency department/pediatric emergency center immediately.
35	Bates et al., 2023 [66]	Ireland	Describing the design, implementation and evaluation of a national bereavement helpline developed as proactive tiered response to immediate be reavement care during the Covid-19 pandemic	Mixed-methods	National Bereavement Support phone Line (BSL)/ Public	April 2020/ 3 h a day, 5 days a week	Responding to anticipated bereavement needs and providing a supportive compassionate listening service, education advice resources and sign posting to community services	Psychological issues	Volunteers reported high levels of job satisfaction (100%) and most of them (86%) expressed an intention to continue volunteering. Providing comfort (93%), educating about grief (81%) and exploring ways of coping with loss (79%), of calls respectively were the most frequent interventions used. The majority of callers had lost a parent followed by loss of a spouse and smaller number had lost a sibling, relative, child or a friend. About 10% of the callers required urgent follow-up, 75.2% of callers were satisfied, 20.7% were neutral and 4.1% were dissatisfied.
36	Gussin et al., 2023 [57]	USA	Analysing of call volume, topics, emotional sentiments, and needs addressed of Covid-19 helpline	Quantitative	Covid-19 helpline/ Nursing home staff	April 2021-April 2022	Promoting a safer nursing home workforce by addressing concerns, providing needed information, encouraging infection prevention activities, as well as symptom reporting, testing, and vaccination.	Vaccination, Covid-19 queries, Psychological issues, Financial and work difficulties	This study supports the value of a confidential helpline for nursing home staff during a pandemic. During Covid-19 surges, staff dominantly expressed fear, anger, and exhaustion. Nearly all inquiries involved requests for information (99%).
37	Ibrahim et al., 2023 [42]	India	Describing mental health concerns and their management among individuals reaching out to the national psychosocial helpline in India during covid-19	Quantitative	Psychosocial helpline/ Public	March 2020/ 24/7	To address the mental health concerns arising out of Covid-19 pandemic	Psychological issues	The helpline has been successful in tackling the concerns of individuals in distress. The concerns of the callers should be characterized into five groups including exacerbation of preexisting mental and physical illness (30.9%), known case of mental or physical illness (19%), Covid-19 related mental health concerns (22.2%), administrative or logistic issues (7.1%), newonset mental health‑related concerns or illnesses (15.9%).
38	Kok et al., 2023 [71]	Uganda	Assessing the functioning of the telehealth approach that was set up to support community health workers during the Covid-19 pandemic	Mixed-method	Toll-free line/ Community health workers	March 2020/ seven days a week, from 7:00 to 23:00 from Monday to Friday, and from 9:00 to 18:00 on Saturday and Sunday	Educating Community Health Workers in rural areas about Covid-19, refering and caring for potential Covid-19 cases, and supporting them in continuing their regular community health work	Covid-19 queries, Psychological issues	The telehealth approach did prove useful for supporting community health workers regular health services in rural communities. The community health workers felt better informed, less isolated and indicated that the support helped them to provide better care. About 77.3% of them answered that the call center support improved the quality of their work.
39	Lai et al., 2023 [47]	China	Tracking the changes in large-sample psychological helpline callers’ mental health concerns over time in China	Quantitative	Ministry of Education-Central China Normal University (MOE-CCNU) Platform/ Public	February 2020/ 24/7	Mental health service	Psychological issues	Callers’ acute stress responses, such as anxiety, fear, stress and somatization decreased while emotions of anger, sadness, and obsession symptoms increased over time. Other chronic reactions, including symptoms of depression, hypochondria, insomnia, and emotions of hopelessness, fatigue, worry, guilt, lasted in the late stage of the pandemic.
40	Munyikwa et al., 2023 [58]	USA	Evaluating the feasibility and preliminary effectiveness of the intervention of virtual call center to address social needs during and beyond the crisis period	Mixed- methods	Virtual Social Needs Response Team (SNRT) call center/ Patients and community members	April 2020/ Two 4-hour shifts per day, 5 days a week	Addressing the exacerbation of underlying economic disparities and mental health concerns for the health system’s patients and employees and experiential learning for third- and fourth-year medical students	Social needs (such as housing and nutrition), Psychological issues	virtual Social Needs Response Team (SNRT) call center has a high level of acceptability and interest among the volunteers. The most common social needs were food assistance (32%), housing (17.4%), and employment (12.7%).
41	Sasidharan et al., 2023 [40]	India	Describing the experiences on UDHAVI helpline’s formation process and report on its function and utility	Mixed-methods	UDHAVI helpline/ public	April 2020/ 3 shifts of 4 h each over a period of 12 h	Establishing a direct link between emergency responders and the general population to address the public’s concerns, communicate accurate information regarding the disease and vaccination, and raise awareness about the current government guidelines, also providing counseling, advice regarding health issues, and referral to other support services	Vaccination, Covid-19 queries	The lines for general information, medical advice, counseling, and logistics support received 25%, 56%, 2%, and 17% calls, respectively. Most of the calls (56%) were related to seeking medical advice, which included queries about vaccinations, pregnancy, Covid-19 transmission, risks, drug interactions. As a result of this initiative, an integrated partnership model proposed for emergency response to any pandemic situation.
42	Tansa et al., 2023 [43]	India	Describing a psychosocial support helpline for health care workers during the Covid-19 pandemic at the National Institute of Mental Health and Neurosciences (NIMHANS)	Quantitative	SAANTHWANA (means consoling people in distress) helpline/ Health care workers	June 2020/ 24/7	Eliminating psychosocial issues of the health care workers, providing them with psychosocial interventions, and creating supportive networking in the hospital	Psychological issues, Social issue, Economic issue, Familial issue	The SANTHWAANA helpline has been able to reach out to the health care workers who required not only administrative support but also psychosocial care. Most of the callers reported psychosocial issues such as distress, social stigma, confusion, lack of motivation, worries about their family, work stress, confusion, sleep disturbance, fear, being unable to look after the family, uncertainty, and lack of concentration on the work and daily activities.
43	Thangarasu et al., 2023 [41]	India	Assessing the profile of calls received in the Covid-19 call center in Tamil Nadu	Quantitative	Covid-19 call center/ Public	March 2020/ 24/7	To help the general public regarding queries related to Covid-19 and other related problems	Vaccination, Covid-19 queries	Maximum calls were received during the year 2021 with morning shift (43.9%) and the majority of calls were from males (80%) between the age group of 16 to 39 years (69.2%). Covid-19 vaccine (65.6%) related calls followed by Covid-19 disease (11.8%) were the most common enquiries made.

### Risk of bias in the studies

The quality and risk of bias in the selected studies were assessed using AXIS [34], MMAT [35], and CASP Checklists [34]. The results are presented in Appendix II. Based on the information provided in each study, most of the studies had a low risk of bias.

### Results of individual studies

According to the finding, most of the studies aimed to describe a Covid-19 hotline/helpline/call center [17, 20, 29, 31, 40, 44, 49, 52, 56, 58–65, 69], analyze the calls [16, 20, 22, 29, 30, 37–43, 45–47, 49–52, 54, 55, 57–68, 71] and the demographic characteristics of the callers [16, 22, 29, 30, 37, 38, 40, 41, 43, 45, 46, 48–50, 54, 58–60, 65, 68, 70], examine the callers’ feedback on the hotline/helpline/call center [30, 31, 36–38, 52, 59, 60, 62, 69, 70], and assess hotline/helpline/call center volunteers’ experiences [53, 66, 71].

**Fig. 2:**
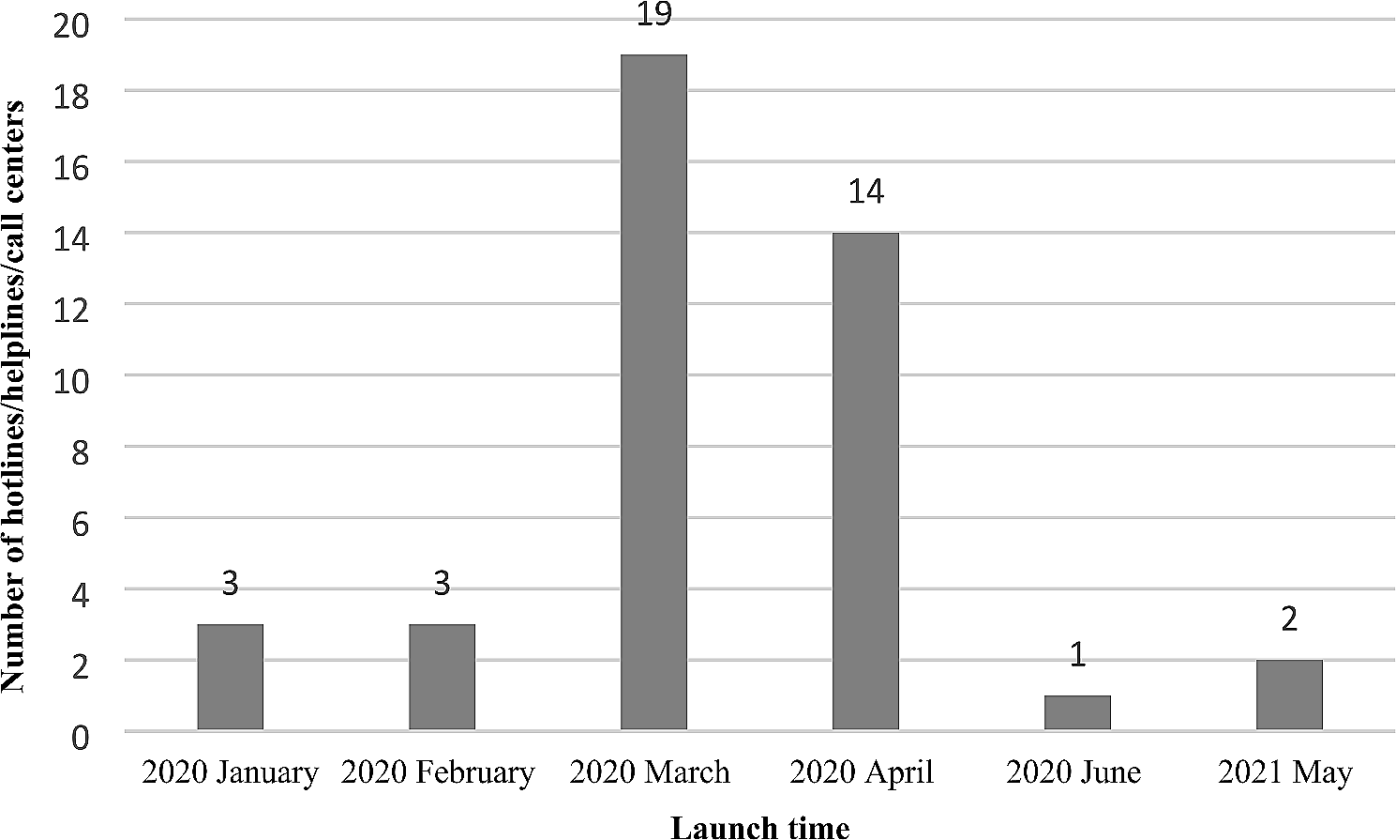
Hotlines/helplines/call centers launch times

In terms of the research methodology, about half of the articles used quantitative methods (*n* = 23), and the rest of them used qualitative (*n* = 3) and mixed methods approaches (*n* = 17). Most hotlines/helplines/call centers were launched in March (*n* = 19) and April (n = 14) 2020, and the others were set up in January 2020 (*n* = 3), February 2020 (*n* = 3), June 2020 (*n* = 1), May 2021 (*n* = 2), and the activation period was not reported in 1 article. (Fig. 2).

### Access time

About half of the hotlines/helplines/call centers (*n* = 22) [29–31, 37, 41–45, 47, 49, 51, 54–56, 60–62, 64, 65, 70, 71] were active seven days a week. The access time for other hotlines/helplines/call centers were different and included six days a week [48], five days a week [16, 58, 63, 66, 68], working days and Saturday [67], Monday to Wednesday [59], (seven days a week, then Monday to Saturday, then Monday to Friday) [20] and the access time was not reported in 12 articles [17, 22, 36, 38–40, 46, 50, 52, 53, 57, 69].

About one third of the hotlines/helplines/call centers were active 24 h a day [29, 30, 37, 41–45, 47, 51, 54–56, 61, 70]. The rest of them were available 16 h a day from Monday to Friday, and 9 h a day on Saturday and Sunday [71],15.5 h a day [52], 14 h on working days and 7 h on Saturdays [67], 12 h a day [16, 20, 31, 38, 40, 49, 50, 64, 68], 9 h a day [62, 63, 65], 8 h a day [58, 63], 6 h a day [55, 60], 4 h a day [39, 59], 3 h a day [66], and the number of hours were not reported in 8 studies [17, 22, 36, 46, 48, 53, 57, 69].

### Hotlines/helplines/call center callers

**Fig. 3:**
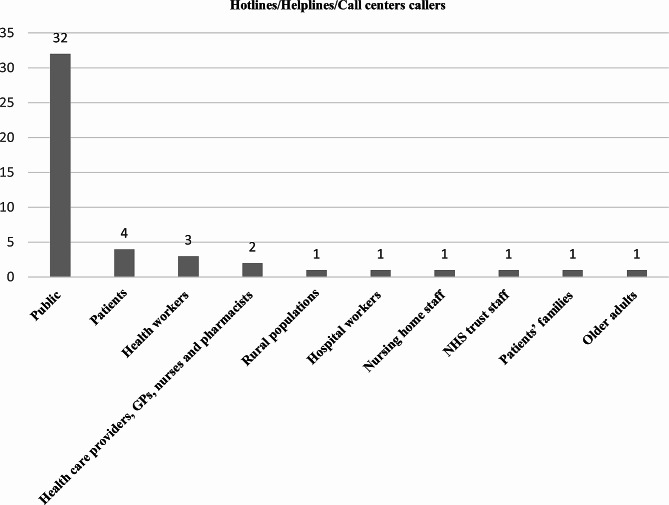
Hotlines/helplines/call center callers

Callers were the public [16, 17, 22, 29–31, 36–42, 44–52, 54, 56, 58–60, 64, 66–69], health workers [43, 69, 71], rural populations [53], hospital workers [61], health care providers, GPs, nurses and pharmacists [20, 62], nursing home staff [57], NHS trust staff [63], patients [20, 55, 58, 69], patients’ families [69], and older adults [65]. (Fig. 3)

### Purpose of implementing hotlines/helplines/call centers

The purposes of using hotlines/helplines/call centers could be categorized in providing psychological support [16, 29, 30, 36–38, 40, 42, 43, 45–48, 50, 58–61, 63, 65, 66, 68, 69], reliable information about Covid-19 [17, 20, 40, 41, 51, 53, 56, 57, 64, 67], consultation [22, 39, 40, 44, 48, 49, 52, 62], and triage services [31, 48, 49, 54, 55, 70, 71].

### Hotlines/helplines/call centeres promotion

As Table 2 shows, in most studies, the Covid-19 hotlines/helplines/call centers were advertised through the media (*n* = 16) including social networks [59, 60], social media [40, 49, 52, 62, 69, 70], media outlets [49, 64], online official social accounts [30], social networking websites [38], Twitter [61], Facebook [16, 61], WeChat public account [44], WhatsApp groups [59], national media [59], TV Channel [39, 49, 70], TV interview [60], news media [44, 52], and media [30, 51]. In some studies, local print media [38], posters [61, 69, 71], newsletters [61], local newspapers [40, 49, 52, 65], news bulletins [16], journalistic notes reporting [59], and flyers [40, 71] were the common ways to introduce the service. In some studies, the service was advertised via the internet pages [30, 63–65] and websites [16, 38, 44, 60, 69], radio stations [30, 49, 52, 65], national radio [66], text message alerts [17, 30], recorded messages [63], emails [61, 63], press conferences [17], broadcasting [30], government announcements [41, 68], organization and administrations [52, 68], nationally known celebrities [66] and health care provider [70].

**Table 2:** Characteristics of Covid-19 hotlines/helplines/call centers in the selected studies

No	Author (Year)	Hotline/helpline/call center promotion	Number of calls	Call agents (operators’) professions	Hotline/helpline/call center provider	Quality of the service
1	Bric and Raile, 2020 [60]	Social networks, the outpatient clinics website, TV	146 calls during 11 weeks (21 March-31 May 2020)	Mental health professionals	Healthcare organization (Psychotherapeutic outpatient clinic of the Sigmund Freud Private University in Vienna)	There is an evaluation link at the clinic’s homepage for the callers. Feedback from the patients were positive.
2	Carson et al., 2020 [53]	Not reported	Not reported	Medical students	Healthcare organization (The University of Nevada, Reno School of Medicine)	After 3 months of hotline implementation, students were asked to complete an anonymous retrospective survey to assess program outcomes and their experiences. Covid-19 hotline improved students’ comfort levels in all areas, with significant changes noted for answering questions, screening, and triaging patients.
3	Geoffroy et al., 2020 [61]	Emails, posters, newsletters, Twitter, Facebook	A mean of 5.73 calls/day	Mental health professionals	Healthcare organization (Assistance Publique–Hôpitaux de Paris)	Not reported
4	Joshi et al., 2020 [36]	Not reported	Each counsellor handled around 70–100 calls per month	Mental health professionals	Other organizations (School of Human Ecology, Tata Institute of Social sciences, India)	Not reported
5	Kristal et al., 2020 [17]	Mayor’s press conferences, City Hall’s Covid-19 text message alerts	More than twenty-five hundred calls per day by mid-March 2020	Nurses, physicians	Healthcare organization (New York City Health + Hospitals)	Not reported
6	Kumar et al., 2020 [62]	Communication bulletins, social media	A total of 310 calls were received in the first 35 days of setting up	Respiratory clinicians (respiratory consultants and a respiratory specialty trainee)	Healthcare organization (Imperial College Healthcare NHS Trust)	Feedback on the effectiveness of the advice line was collected from general practitioners who used the service, and they were satisfied with the service (mean score of 9.3/10).
7	Margolius et al., 2020 [54]	Not reported	10,112 patients called the hotline during 5 weeks	Nurses, physicians	Healthcare organization (MetroHealth, health care system in Cuyahoga County in Northeast Ohio)	Not reported
8	Matthewson et al., 2020 [63]	Trust intranet page, email	655 calls were received within the first 4 weeks of implementing the telephone support service	Mental health professionals	Healthcare organization (National Health Service (NHS) occupational health services (OHS))	Informal positive feedback was received from managers. Mean waiting time for callers to be put through to a call handler was 28 s indicating quick response times. Initial informal feedback suggested that access to clear, timely information was useful to staff in a healthcare setting.
9	Pelicon et al., 2020 [64]	Press conferences, media outlets, Internet	More than 40 thousand calls received in its first ten weeks.	Medical students, integrated professionals	Government and a healthcare organization (The Government Communication Office of the Republic of Slovenia in cooperation with the Clinic for Infectious Diseases and Febrile Illnesses of the University Medical Centre Ljubljana, the National Institute of Public Health, the Ministry of Health and the Ministry of Public Administration.)	Not reported
10	Ravindran et al., 2020 [37]	Not reported	20,475 calls answered in the first month (April 2020).	Mental health professionals	Government and a healthcare organization (The Ministry of Health and Family Welfare (MOHFW), Government of India in collaboration with the National Institute of Mental Health and Neurosciences (NIMHANS))	Getting preliminary feedback from the callers. Over 90% of the callers were satisfied with the provision of the service.
11	Shao et al., 2020 [44]	Website, WeChat public account, news, media	59,495 telephone hotline conversations between January 23, 2020, and March 15, 2020.	Nurses, physicians	Healthcare organization (Taizhou Hospital and Enze Hospital)	Not reported
12	Abdelghaffar et al., 2021 [69]	Displaying posters all over the hospital in all departments, social media, the official website of the Hospital	Not reported	Mental health professionals	Healthcare organization (Mongi Slim hospital in the Tunis)	There was widespread positive feedback from patients and professionals concerning the services.
13	Abdullah et al., 2021 [29]	Not reported	Not reported	Mental health professionals	Healthcare organization (The Department of Psychiatry at Westchester Medical Center Health Network in Valhalla, New York)	Not reported
14	Cheng et al., 2021 [31]	Not reported	More than 5825 calls were between March 30 and June 1, 2020.	Nurses, medical students	Healthcare organization (Oregon Health & Science University (OHSU))	86% of patients reported that their questions were answered during the call, and 90% said they would recommend this service to a friend or family member.
15	Cher et al., 2021 [55]	Not reported	An average of 91 calls per day	Nurses, medical assistants, patient service representatives, medical students	Healthcare organization (Michigan Medicine, the academic medical center affiliated with the University of Michigan)	Not reported
16	Du et al., 2021 [30]	Internet, media, and radio stations, text messages (online official social account, and broadcasting)	A mean of 37.10 calls per day	Mental health professionals	Healthcare organization (Mental health center of the city, the fourth people’s hospital of Chengdu)	The effectiveness of the hotline was evaluated through verbal feedback from the callers. A total of 95.0% agreed that the hotline helped them overall.
17	Hazarika et al., 2021 [38]	Assam Police website, local print media, social networking sites	Not reported	Mental health professionals	Government and a healthcare organization (The Police Department of the Government of Assam in collaboration with the Department of Psychiatry, Gauhati Medical College Hospital (GMCH))	Most of the callers were satisfied (49.8%) and appreciated (42.3%) the service.
18	Iqbal et al., 2021 [50]	Not reported	201 calls in the first month of operation.	Not reported	Healthcare organization (Between three well-established mental health service providers (Brac Institute of Educational Development, Psychological Health and Wellness Clinic (PHWC) and Kaan Pete Roi)	Not reported
19	Jang et al., 2021 [20]	Not reported	A total of 2548 calls over 7 weeks.	Radiology technologists, central scheduling staff, assistant radiology technologists, senior radiology residents	Healthcare organization (A large academic tertiary care center)	Not reported
20	Meaden et al., 2021 [56]	Not reported	Over 35,000 calls between January 27, 2020, to May 31, 2020.	Nurses, pharmacists, physicians, physician assistants, students in medicine, pharmacy, nursing, dentistry, and public health	Government and a healthcare organization (The State Department of Health and the New Jersey Poison Information and Education System (NJPIES))	Not reported.
21	Wahl et al., 2021 [65]	Local newspapers, radio stations, homepage of the city of Mannheim	A total of 55 older adults called duringthe initial stage of the pandemic (13 April–15 June).	Mental health professionals	Healthcare organization (Central Institute of Mental Health, Mannheim (CIMH), University of Heidelberg)	Not reported
22	Zabrzygraj and Świtaj, 2021 [16]	News bulletins of the Polish Televi sion TVP Info, website, facebook	A total of 262 individuals contacting the helpline from March to July 2020.	Mental health professionals, social worker	Healthcare organization (Warsaw-based Mokotow District Mental Health Center)	Not reported
23	Alfatih and Rachmawati, 2022 [51]	Media	The number of calls was 2769 from March 14, 2020, to March 29, 2021,	Not reported	Government and a healthcare organization (The Regional Disaster Relief Agency (BPBD) and the Health Office of DIY)	Not reported
24	Egić, 2022 [67]	Not reported	Not reported	Physicians, mental health professionals	Government (The initiative of the “Novi Sad” Mayor)	Not reported
25	Khan et al., 2022 [39]	TV Channel	Not reported	Physicians	Government and a healthcare organization (Aaj Tak News Channel and AIMS2Health Private Limited)	Not reported
26	Monreal-Bartolomé et al., 2022 [68]	Different administrations and entities	A total of 1411 calls were answered in the 46 days that the service was active, with an average of 30.67 calls/day.	Mental health professionals	Government and a healthcare organization (The Professional College of Psychology of Aragon together with the Department of Health of Aragon and with collaboration from the Rey Ardid Foundation)	Not reported
27	Nina-Mollinedo et al., 2022 [22]	Not reported	A total of 167,261 calls were received in the first 100 days of its implementation (March-June).	Physicians	Government (The Bolivian Ministry of Health and Sports)	Not reported
28	Ouyang et al., 2022 [45]	Not reported	A total of 4319 calls were received from 27 January to 30 June 2020.	Mental health professionals	Healthcare organization (The Jiangsu Psychological Crisis Center in Nanjing Brain Hospital affiliated with Nanjing Medical University)	Not reported
29	Peng et al., 2022 [46]	Not reported	706 people sought psychological assistance from January 25 to June 23 2020	Mental health professionals	Healthcare organization (The Psychological Crisis (Intervention Professional Committee of Guangdong Mental Health Association))	Not reported
30	Singh et al., 2022 [52]	Social media, news media, radio stations, newspapers, organizations that were actively working on Covid-19 management, local government bodies, and large organization networks	Total number of Incoming calls from May 2021 till February 2022 was 12,555.	Physicians, medical officers, nurses	A non-profit organization (ASK Foundation (a non-profit organization based in Nepal) with help of a private telecommunication company)	Patients had a very positive response regarding the service.
31	Sosa Lovera et al., 2022 [59]	A written advertisement containing the telephone numbers and schedules of the volunteers, and short videos promoting the service, by the social networks of the University and the Dominican College of Psychologists, WhatsApp groups and personal social networks	Over the course of four months, the programme assisted 497 people.	Mental health professionals	Healthcare organization (The School of Psychology at the Autonomous University of Santo Domingo (UASD))	Feedback was collected and service assessment surveys were completed by care staff and people who had received care through the helpline.
32	Abdelbaky et al., 2023 [70]	Social media, T.V, Health care provider	Not reported	Nurses, physicians	Governmental organization (Ministry of Health)	Almost about 75.1% were positive and 50.1% were satisfied with using the hotline emergency triage services.
33	Alabdulla et al., 2023 [48]	Not reported	A total of 12,594 calls were answered during the 12-month study period.	Mental health professionals, triage clinicians	Government and a healthcare organization (The Hamad Medical Corporation Mental Health Service, the Ministry of Public Health and the Primary Health Care Corporation)	A patient satisfaction survey, demonstrated predominantly positive feedback and 90% of callers stated that they would recommend the service to a friend or a relative.
34	Arafa et al., 2023 [49]	Media outlets (newspapers, television, radio), social media platforms	A total of 60,229 calls were received between April-August 2020 (during the five months).	Physicians	Healthcare organization (Hamad Medical Corporation, the public health provider in Qatar)	Not reported
35	Bates et al., 2023 [66]	The line was widely advertised through the health service, promoted on national radio and supported by nationally known celebrities.	In total, 691 calls were recorded on Amazon Connect.	Volunteers were graduates of the Royal College of Surgeons in Ireland, graduates of a postgraduate training in Bereavement	Government and a healthcare organization (The Irish Hospice Foundation (IHF) and the Irish National Health Service Executive (HSE))	Volunteers expressed a high level of satisfaction with the management of the line, and the training provided. They reported high levels of job satisfaction (100%) and most (86%) expressed an intention to continue volunteering. 75.2% of callers were satisfied, 20.7% were neutral and 4.1% were dissatisfied.
36	Gussin et al., 2023 [57]	Not reported	The median number of monthly inquiries was 22	Not reported	Healthcare organization (University of California Irvine School of Medicine)	Not reported
37	Ibrahim et al., 2023 [42]	Not reported	A total of 169 valid callback entries were reviewed from March 29, 2020 till May 31, 2020.	Mental health professionals	Government and a healthcare organization (The behest of the Ministry of Health and Family Welfare, Government of India, in coordination with the National Institute of Mental Health and Neurosciences, Bengaluru, India)	Not reported
38	Kok et al., 2023 [71]	In the first two weeks, all community health workers were contacted by the call-center to make them aware of the support that they could get and to provide them with information about Covid-19. To further promote the use of the call center and share information about CO VID-19, flyers and poster were printed and distributed among all CHWs	A total of 35,553 calls to the call center was received between March 2020 and June 2021.	Nurses, physicians, clinical officers	Governmental and a healthcare organization (Collaboration with the Ministry of Health Uganda, and a local consortium)	In-depth interviews were conducted with the staff of the call-center and Community Health Workers who had used the call center. The telehealth approach did prove useful for supporting community health workers regular health services in rural communities.
39	Lai et al., 2023 [47]	Not reported	26,870 calls were received from February 28, 2020, to April 23, 2021.	Mental health professionals	Governmental organization (The Ministry of Education in China)	Not reported
40	Munyikwa et al., 2023 [58]	Not reported	Virtual call center served 1727 patients and community members From April 2020 to March 2022.	Medical, Nursing, and Social Work Students	Healthcare organization (The University of Pennsylvania and its accompanying health system (UPHS))	Feedback was obtained through semi-structured interviews with students, patients, and health care providersand it was largely positive.
41	Sasidharan et al., 2023 [40]	Various media, including websites, social media platforms, and local newspapers. Flyers in both English and Tamil were also used.	A total of 677 calls to the helpline was received between mid-May and mid-June 2021.	Volunteers among the medical and nonmedical personnel, as well as the support staff (medical consultants, nurses, physicians, chaplains, Mental health professionals)	Healthcare organization (A tertiary care institution in India)	The volunteers reported that the team evaluations, feedback, counseling, and coordination among the teams from different areas helped them feel supported through the experience while making an impact.
42	Tansa et al., 2023 [43]	Not reported	327 calls were received between June 2020 and March 2021.	Mental health professionals	Healthcare organization (The national Institute of Mental Health and Neurosciences, Bengaluru, India)	Not reported
43	Thangarasu et al., 2023 [41]	The telephone number for the call center was distributed by the government announcements.	278,665 calls were received during the period of November 2020 to June 2023.	All of the employees from the Directorate of Public health and preventive medicine with advanced degrees and training in different fields of healthcare delivery	Government (The Tamil Nadu government initiated the call center)	Not reported

### Call agents’ (operators’) professions

The profession of the hotlines/helplines/call centers agents (operators) in selected studies were mental health professionals (*n* = 20), such as psychologists, psychiatrists, psychotherapists, psychological practitioners, psychiatry residents, psychological consultants, psychotherapy professionals, clinical psychologists and neurologists; clinicians (*n* = 18) such as physicians, consultant physicians, physician assistants, triage physicians, general practitioners (GPs), medical assistants, medical consultants, medical officers, clinical officers, respiratory clinicians, triage clinicians; nurses and psychiatric nurses (*n* = 12); radiology technologists, assistant chief radiology technologists, and senior radiology residents (*n* = 1); students (*n* = 9) in medical, medicine, pharmacy, nursing, dentistry, public health, and social work; social worker and psychiatric social workers (*n* = 2); pharmacists (*n* = 1); volunteers graduates in postgraduate bereavement courses (*n* = 1); epidemiologist (*n* = 1); chaplains (*n* = 1); nonmedical staff, central scheduling staff, and patient service representatives (*n* = 3). In 20 studies, the profession of call agents were not reported.

### Hotlines/helplines/call centeres providers

As Table 2 shows, Covid-19 hotlines/helplines/call centers were implemented by healthcare organizations (*n* = 25), government (*n* = 5), cooperation between government and healthcare organizations (*n* = 11), a non-profit organization (*n* = 1), and other organizations (*n* = 1).

#### Evaluation of the service quality

In 15 studies, service quality was evaluated by receiving feedback from the callers and operators. Overall, the callers and operators were satisfied with Covid-19 hotlines/helplines/call centers services in most studies. (Table 2).

### Challenges of the Covid-19 hotlines/helplines/call centers

The most common challenges mentioned in the articles included unavailability of hotlines/helplines/call centers [44, 52, 60, 62, 70], lack of specific protocols to support different situations of callers [47, 59, 60], delay in updating information about Covid-19 [36, 51, 53, 58, 62], different backgrounds and experiences of hotlines/helplines/call centers volunteers [37, 45, 47, 53], and lack of experience or the previous models for developing and implementing hotlines/helplines [53, 60]. Moreover, the physical examination of patients was not possible [17, 29, 37, 52, 55, 57, 61, 70, 71], and there were challenges related to human resources and sufficient equipment [51, 52], delay in receiving a prompt response due to the operators’ workload [51, 69], receiving calls via personal phones and being exposed to threats such as hacking their accounts [59, 69, 70], inadequacy of training [53, 58, 66], limited funds [69], and a large number of calls [56, 70]. A summary of challenges is presented in Table 3.

**Table 3:** Summary of the challenges and lessons learned in the selected articles

No	Author, Year	Challenges	Lessons learned
1	Bric and Raile, 2020 [60]	- Had no previous experience or models to orient them to set up the helpline. - Setting up a telephone number and connect the psychotherapists and psychologists, who were not allowed to go out due to the lock-down, and therefore had to make the telephone calls in their home office. - Criticism of some psychotherapists was related to the lack of clarity in the initial phase of the helpline regarding the time of availability. - Many put down their help offering due to the request to document their contacts in the context of crisis calls. - There were more questions about why they had not received any assignments.	For future projects, even under extreme time pressure, the feedback should first be received by the participants to improve and any queries should be answered before the initiative is launched.
2	Carson et al., 2020 [53]	- Challenges for the hotline included rapidly evolving information about SARS-CoV-2 and the varying levels of clinical experience among the volunteers. - When developing the hotline and associated curriculum, there was limited literature available for reference on similar audio-only trainings for students in a triage capacity.	The remote clinical experience utilizing telehealth (Covid-19 hotline) and weekly e-trainings is a viable and effective educational model to develop medical students’ clinical exam and decision-making skills.
3	Geoffroy et al., 2020 [61]	The hotline could not make a formal diagnosis.	Mandatory factors for the implementation of a hotline include a clear mandate, the adequate and appropriate human resources (volunteers), a functional technology platform, ensuring anonymity, as well as a clear communication plan (sending regular reminders about the existence of 24/7 hotline for hospital workers).
4	Joshi et al., 2020 [36]	The challenges experienced by counsellors included technology issues, linguistic diversity, constant exposure to crisis stories, the dearth of resources in the community and difficulties experienced in the personal lives.	Not reported
5	Kristal et al., 2020 [17]	- Evaluating a caller’s respiratory status and risk for sudden decompensation. - Evaluating low oxygen saturation without clinical manifestations over the phone.	- Tools such as the Roth score, were proposed, to evaluate shortness of breath telephonically, and where feasible, the provider could utilize video platforms to visually inspect the caller’s respiratory status. -Providing high-risk patients with a pulse oximeter, so they could monitor their oxygen saturation at home. - Priority should be given to promoting it as early as possible to vulnerable communities.
6	Kumar et al., 2020 [62]	- Availability of the respiratory specialists (job planning). - Streamline information flow and appropriate time management if service usage was high. - Ensuring appropriate governance of the system, risk reporting and record keeping, which may be challenging if joint records systems between primary and secondary care are unavailable. - Uncertainties when providing advice related to Covid-19 due to the rapidly evolving evidence.	Not reported
7	Margolius et al., 2020 [54]	Not reported	Not reported
8	Matthewson et al., 2020 [63]	Not reported	Services should be changed over the course of the pandemic based on the feedback and demand from the staff members.
9	Pelicon et al., 2020 [64]	Not reported	When developing a crisis response system, a centralized, easily accessible, toll-free, and well-promoted telephone helpline should be established for providing reliable and trust-worthy information given by competent advisers in cooperation with various experts and supported by psychologists and psychiatrists.
10	Ravindran et al., 2020 [37]	- Being unable to see a distressed individual face-to-face. - Getting calls concerning logistical issues and not being able to help directly with their resolution. - Various backgrounds and experiences of volunteers in mental health. - Individuals with acute psychiatric emergencies posed difficulty to address over a phone call.	Not reported
11	Shao et al., 2020 [44]	- Patients’ low awareness of the Covid-19 special line. - The timeliness of admissions when the number of waiting patients in each channel exceeded 3.	Not reported
12	Abdelghaffar et al., 2021 [69]	- Volunteer psychiatrists and psychologists provided the psychological support for free and in addition to their routine work. - Volunteers received the calls on their personal phones. - The psychological support unit had no budget from the hospital. -There was a stigmatization of seeking psychiatric help in the Tunisian culture.	Not reported
13	Abdullah et al., 2021 [29]	Due to the nature of the hotline calls, which were not like formal clinical encounters, a limited clinical framework for the evaluation of symptom were used.	Mental health services must be adequately staffed and prepared to screen and treat those individuals who needed assistance. The framework of this hotline service could be successfully adapted by others, and that institutions across the country should further develop easily accessible tele-mental health services at no cost to minimize emergency department visits and appropriately triage psychiatric care based on acuity.
14	Cheng et al., 2021 [31]	Not reported	Having adequate resources and funding available, it is possible to rapidly implement a multiphase coordinated approach to aid primary care teams on a statewide level to respond to a pandemic.
15	Cher et al., 2021 [55]	- A lack of physical exams, which were often necessary to properly evaluate the cardiopulmonary complaints - Maintaining social distancing standards and properly sanitizing shared workspaces	Although imbalances between health care supply and demands are likely to arise during future pandemics or subsequent waves of the Covid-19 pandemic, call centers and hotlines can be used to reduce the burden placed on the strained medical systems.
16	Du et al., 2021 [30]	Not reported	Not reported
17	Hazarika et al., 2021 [38]	The old system of accessing healthcare services physically might be so ingrained in the community that the benefit of teleconsultation might not reach a majority of the population who needed care due to their ignorance of teleservices as a satisfactory option, hesitation to take help from someone with whom they had no previous interaction, poor knowledge and understanding of the technological advancement like video conferencing.	This modality of intervention could be an effective way of providing mental healthcare in lower and middle-income countries (LMIC) where resources are limited.
18	Iqbal et al., 2021 [50]	Not reported	Not reported
19	Jang et al., 2021 [20]	Not reported	Not reported
20	Meaden et al., 2021 [56]	- Because of overwhelming call volume, it was necessary to increase our overall phone system capacity, staffing, and remote capabilities to both meet the demands for coronavirus calls and fulfill the center’s obligation to provide poison information and exposure management.	- Call centers should ensure appropriate staff education on current events and public health guidance so that they may communicate accurate information to callers. - Language is of particular importance with interacting with the public through a call center; as such, it is important to have interpreters available to assist healthcare professionals on the line. - Up-to-date knowledge of current events and recommended public health guidance is necessary to best equip hotline staff to field questions and offer expert advice
21	Wahl et al., 2021 [65]	Not reported	Not reported
22	Zabrzygraj and Świtaj, 2021 [16]	- Receiving calls from people who experienced domestic violence in the absence of the perpetrator or at nighttime - People threatened by domestic violence may have tended to choose helplines specifically targeting their needs (e.g., Blue Line, emergency helplines).	Not reported
23	Alfatih and Rachmawati, 2022 [51]	- The distribution of information updates from the center was often slow. The information was related to Covid-19 handling policies, both at the national and regional levels. - Limited human resources and the lack of adequate equipment. - The workers served as operators when they were doing their jobs in different departments. So, some calls and messages could not be served immediately. - Calls and messages often piled up due to only one smartphone device with the WhatsApp application to serve various community questions regarding Covid-19. - The slow response from the operator.	- Adding more equipment or the procurement of a call center that could be integrated with different parts of a health center, hospital, or other agencies. - It is necessary to establish a permanent management structure for hotlines, so that in practice the hotline officers can focus more on managerial issues. - There is a need for innovation in the use of ICT, for example by integrating hotlines with existing smart city applications.
24	Egić, 2022 [67]	Not reported	Not reported
25	Khan et al., 2022 [39]	Not reported	Not reported
26	Monreal-Bartolomé et al., 2022 [68]	- Not all the calls could be answered and not all the answered calls were registered, since their registration was something that the psychologists offered to do voluntarily.	Not reported
27	Nina-Mollinedo et al., 2022 [22]	- The number of calls received and taken by the call center turned out to be significantly higher than the number of the teleconsultations completed for patients with suspected Covid-19 who were referred. - There were missing data due to the sociocultural limitations of target population, and in some cases, the instability of connectivity. - The impossibility of transferring medical devices due to the logistical and legal reasons caused carrying out the task to the patient’s homes.	Not reported
28	Ouyang et al., 2022 [45]	- The public demand for psychological services increased the professional requirements for hotline operators, who need to have corresponding professional qualifications and rich experience in helping others.	- It is necessary to establish a talent pool because usually the psychological hotline operators have basic psychological knowledge and basic counseling skills. - It is necessary to improve the supervision and training system for professionals and form a transparent management system for the operation of a psychological hotline.
29	Peng et al., 2022 [46]	Not reported	Not reported
30	Singh et al., 2022 [52]	- Having a shorter (three-digit) number could have improved the number of incoming calls. - Medical consultation was provided using audio, and this had its challenges, especially in objectively evaluating a patient’s condition. - This service was only available during day time. - The language was likely another barrier to the expansion of the service across Nepal as the services were advertised in English, which is not the language spoken by the majority.	- The accessibility of the services could be expanded to 24 h., if additional financial assistance was received and, more healthcare professionals were recruited.
31	Sosa Lovera et al., 2022 [59]	- The availability of technological resources for the development of the helpline and the vulnerability of the personal data of the team. - The professionals were using private numbers and they were exposed to threats such as hacking their WhatsApp accounts. - Limited Internet access or restricted availability of free calls. - Call or chat interruptions (only staff who had funds available on their phone could return the call and continue the intervention). - There was no specific protocol for supporting the situations of violence or caring for people at risk of suicide. - The helpline management team’s efforts to establish collaboration with government entities were not successful.	- This therapeutic modality was cost-effective and helped to reduce the gap in mental health care. - The professional competencies in mental health care issues in emergencies and disasters, and the experiences of approaching digital mental health need to be considered. - Leadership and organizational skills of the team granted the development of the idea in a short time.
32	Abdelbaky et al., 2023 [70]	- Very busy during calls. - Misunderstanding of the remote hotline treatment by Covid-19 patients - Wrong initial assessment. - Inability to physical exam or actual face to face communication. - Medical error (error in treatment and advice). - Services can jeopardize patient privacy.	- Providing high standard education and training for critical care nurses and doctors about telehealth will aid in improving the quality of remote triage services. - Public awareness of remote hotline emergency triage calls services should be increased.
33	Alabdulla et al., 2023 [48]	Not reported	Diverse and culturally competent team members need to communicate in Arabic, English, Hindi, Urdu, Malayalam and Pashtu.
34	Arafa et al., 2023 [49]	Not reported	- With adequate resources and better coordination, the service setup as well as its operations ran smoothly, and the triage physicians as well as the specialized physicians were able to deliver Urgent Consultation Centre’s objectives. - The setting up and smooth functioning of hotlines require a complex web of interactions, and intersectoral and interdepartmental collaboration between various ministries, hospitals, institutes, departments, and specialties. - Telephone advice depends on an appropriately skilled workforce who is accessible to sort out and settle calls over the phone, with attention to patients’ emotional and medical needs.
35	Bates et al., 2023 [66]	Some volunteers felt they needed more training.	-The daily debriefs of volunteers with management and weekly supervision is helpful including discussing and listening to feedback from the line coordinator as well as the other volunteer on the line in debrief session.
36	Gussin et al., 2023 [57]	Emotional sentiment was classified by subjective assessment during each call.	- Helpline services should be advertised to all nursing homes in the county. - Assistance was offered in English and Spanish by two trained responders. The utility of the helpline services was enhanced by providing support in both English and Spanish to address common primary languages among nursing home staff in Southern California.
37	Ibrahim et al., 2023 [42]	Not reported	- The effectiveness of the helplines needs to be assessed and monitored at frequent intervals. - Regular feedback from the service providers and users will help such services to be more reliable and helpful to the community in future.
38	Kok et al., 2023 [71]	- Community health workers were used to work independently, and the lack of physical interaction made it difficult to promote the call center. - As some community health workers did not use the call center, it was the general skepticism about telephone services.	- Those who consider using telehealth to support community health workers elsewhere can examine to what extent similar circumstances exist and how telehealth can best be set up to contribute to health in a specific local situation - The telehealth approach could be set up and scaled up in a short period and appears to be a low-cost strategy for providing useful and flexible support to community health workers in rural communities.
39	Lai et al., 2023 [47]	- Although the counselors were qualified and had rich experience, their reports of the callers’ mental health concerns may be biased. - The measurements were forced to have binary and non-standardized questions because the data collection needed to be short and straightforward without influencing the counselors’ main works. The measurement formats may impact the reliability and stability of the outcomes.	- The large-sample data of psychological helplines represent the public’s psychological needs, indicating what problems should be focused on. The findings can help the government and professionals provide better mental health services and adjust policies and interventions in different pandemic stages.
40	Munyikwa et al., 2023 [58]	- Volunteers revealed several challenges, including managing volunteer comfort with the techniques taught during orientation, managing emotional difficulties when confronting the challenges that patients had, and making resource guides easy to navigate during conversations with clients. - Consolidating and updating social resources tools posed a significant challenge in the early days, which required significant time and in-kind effort from several volunteers to maintain an active and accurate database of rapidly evolving local resources. - They faced significant challenges in recruitment, training, and retention.	- Ongoing sustainability plans also include advocating for policies that facilitate insurance reimbursement and the development of creative partnerships with insurers and foundations to either incentivize or fund such services.
41	Sasidharan et al., 2023 [40]	Not reported	- The team evaluations, feedback, counseling, and coordination in different areas helped the volunteers feel supported through the experience while making an impact. - When employees feel supported and have reliable information, there will be enough traction for administrative teams to request volunteers to respond to the pandemic. - The training provided by the hospital to the volunteers ensured that all callers received validated medical advice. - Mock sessions contributed to increasing confidence in the delivery of appropriate services and familiarity with using the apps and electronic devices - Before the UDHAVI initiative was implemented, a continuing medical education program was conducted to ensure the use of evidence-based protocols for Covid-19 management at the hospital. -- Ensuring protocols were evidence based and in compliance with the national and state guidelines, and flexibility in the protocol development easily ensured scalability and deployment. - It is critical to promote the helpline to ensure that individuals had access to information, support, and resources during the pandemic. To reach as many people as possible, various media, including websites, social media platforms, and local newspapers were used. Also, flyers in both English and Tamil was created and displayed at all campuses of the hospital and its rural units. - An integrated partnership model based on the UDHAVI helpline was developed that could be used for future emergency response in any pandemic or epidemic (The model has 4 phases with 8 strategies).
42	Tansa et al., 2023 [43]	Not reported	Not reported
43	Thangarasu et al., 2023 [41]	Not reported	- Every district and every state should have a dedicated call center to address the problems of the public. - In addition to the regular call centers there should be additional dedicated call centers to the public at times of epidemics and emergencies similar to the hotlines available for disaster by national disaster management authorities. - There should be some calculations of the time taken to collect information from the public and the time taken to address the problem to assess the efficiency of the information providers. - This call center should be incorporated with the Disaster Management Authorities at times of natural disaster to provide more support to the public in reducing their sufferings. - More dissemination of information regarding the call centers functioning, working hours and the information shared through it should be collected to improve the access by all the people from highly developed cities to poorly developed villages.

#### Lessons learned

Hotlines/helplines/call centers can be used to reduce the burden placed on the strained medical systems, especially in lower and middle-income countries (LAMIC) [38, 55]. Essential factors for successful hotline/helpline/call center development included clear instructions for implementation [40, 61], adequate and appropriate human resources [31, 49, 61], receiving support by competent advisers [45, 49, 59, 61], adequate technological resources [31, 49, 61], getting the feedback from users of the hotline [40, 42, 60, 63, 66], forming a transparent management system [45], staff training [40, 53, 56, 70], adequate funding [31], availability of interpreters [48, 56, 57], maintaining anonymity [61]. a clear communication plan [61], accessibility [52, 64, 71], toll-free [52, 64, 71], updating information [41], promoting the helpline [40, 41, 57, 64, 70], and integrating the call center/hotline with different parts of a health center or hospital [51]. Another issue is related to the 24 h accessibility of this services which needs more contribution from the healthcare professionals and additional financial assistance [52]. A summary of challenges and the lessons learned is presented in Table 3.

#### Syntheses

The results showed that, in terms of the geographical distribution, most studies were undertaken in Europe & Central Asia (*n* = 10), South Asia (*n* = 10), and North America (*n* = 10). In terms of the income, about half of the studies were conducted in high income countries (*n* = 21), 14 in lower middle-income countries, 7 in upper middle-income countries, and 1 in a low-income country. Half of the hotlines/helplines/call centers were active seven days a week, and about one third of them were active 24 h a day. About half of the hotlines/helplines/call centers provided psychological services and the rest of them provided Covid-19 information services as well as answering questions, consultation, and triage services. In the selected studies, the challenges such as the unavailability of services, lack of up-to-date information, limitations in the physical examination of the callers, lack of budget allocation, and lack of a standard protocol were identified. To achieve success, hotlines/helplines/call centers need to be easy to use with free access. They also need to be supported by proper advertising of the hotlines/helplines/call centers, adequate human resources, training, funding, equipment, and receiving feedback.

## Discussion

This systematic review aimed to investigate the characteristics of telephone lines and call centers implemented in various countries during the Covid-19 pandemic, along with their associated challenges and lessons learned. The research findings revealed that most studies initiated the deployment of hotlines/helplines/call centers in March and April 2020. These channels were established to deliver psychological services, Covid-19-related information, consultation, and triage services. The primary challenges highlighted in the examined studies encompassed restrictions on physical examination of patients, unavailability of hotlines/helplines/call centers, exposure to rapidly evolving Covid-19 information, inability to promptly respond to incoming calls, various clinical backgrounds and experiences of volunteers who worked as a call agent (operator), as well as the lack of established protocols and previous successful models for telephone line implementation. The lessons derived from these studies demonstrated that several key factors need careful consideration before implementing hotlines/helplines/call centers. These factors encompass ensuring ease of use and free access, ensuring an adequate number of competent human resources, providing proper staff training, allocating sufficient financial resources, procuring additional equipment, utilizing video tools and platforms to enable visual assessment of callers, ensuring access to interpreters, and establishing mechanisms for receiving feedback from relevant stakeholders.

Utilizing remote health services experienced a substantial increase in the year 2020 when the global Covid-19 pandemic started [72]. In the early stages of the lockdown measures, health authorities recommended the replacement of in-person counseling sessions with remote consultations in order to minimize the risk of viral transmission within the traditional clinic settings [73]. Koonin et al.‘s study demonstrated that the provision of healthcare services underwent a transformation during the Covid-19 pandemic, as evidenced by a 154% surge in the utilization of remote health services during the last week of March 2020 compared to a similar timeframe in 2019 [74]. It appears that the World Health Organization’s global declaration on March 11, 2020 has exerted a significant influence on public behavior regarding the adoption of remote health options [75]. Similarly, Wong et al. discovered that the United States exhibited the highest demand and interest in offering remote health services compared to other countries [75].

The results of a systematic review on the telemedicine platforms in lockdown periods during the Covid-19 pandemic indicated that the real-time interaction modalities, for example, online chatting, telephone communication, and video conference technologies offered immediate and easy-to-use services, and provided care remotely during the current pandemic. This method of communication was more effective than other methods, such as E-mail, fax, forums, file transfer technologies, and prerecorded multimedia [76]. Similarly, the short message service (SMS) can timely provide abundant anti-epidemic alerts to mobile users. SMS tracking platforms could be useful as an early warning system to refer patients with worsening clinical status to hospital-based care or additional clinician advice [77]. Yu et al.’s study showed a positive attitude towards content and the necessity of public-interest in SMS during the pandemic [77].

Overall, developing free interactive chat services can help the general public learn about important issues related to Covid-19. Users can ask questions and get connected to appropriate healthcare services for Covid-19 [78]. Different researchers reported positive experiences delivering care remotely using both telephone and video during the Covid-19 pandemic and believe both modalities are critical for enabling access to care [79]. Video-specific advantages included enhanced ability to engage patients and use of visual cues to get a comprehensive look into the patient’s condition. Similarly, telephone presented unique benefits, including greater privacy, feasibility, and ease of use [79]. The telephone is a familiar and dependable technology, which is adequate for many Covid-19 related conversations. Patients who just want general information about Covid-19 should be directed to a telephone message or other online resources [80].

Research investigating the impact of Covid-19 has revealed that apart from its mortality consequences, this novel disease has triggered various psychological effects, including heightened levels of anxiety. Fardin’s study showed that as the prevalence of Covid-19 and the subsequent imposition of restrictive measures mounted, anxiety levels were also escalated. Consequently, enhancing public awareness about this disease and broadcasting psychological programs focused on stress management through media outlets and contributed to anxiety reduction within communities [81]. Furthermore, quarantine measures have been found to inflict significant adverse psychological effects on individuals, including post-traumatic stress symptoms, confusion, and anger [82].

In a comprehensive review study conducted by Pedrosa et al. the impact of Covid-19 on global mental health was examined and the results revealed that not only are vulnerable groups affected by the transmission of the virus and resulting fatalities, but they also encounter emotional, behavioral, and psychological consequences such as fear, anxiety, depression, and suicidal thoughts [83]. Similarly, in the present study the results showed that, seeking remote psychological services was one of the main purposes of contacting hotlones/ helplines/call centers.

The results of the present study showed that one of the objectives of implementing phone lines and call centers was providing information related to Covid-19 and answering questions. As it was a new disease, providing the callers with sufficient information about the virus was necessary, while preventing the dissemination of incorrect information [84]. In this regard, Alvarez-Risco et al. found that the Covid-19 pandemic led to an increase in the sharing of fake news and misinformation from unofficial resources. This, in turn, can be detrimental to the control of the pandemic and hinder the provision of appropriate responses by public health authorities [85].

One way to prevent the spread of misinformation is to provide reliable information resources. Central and local governments play a role similar to that of policy-makers in providing Covid-19-related information. This need might be met via using information and communication technologies, as well as establishing call centers and hotlines/helplines [51]. The Covid-19 helpline is an interactive information service that allows individuals to directly contact and obtain information about Covid-19 through the phone. The use of the Covid-19 helpline can be an option for the general public to receive information more quickly, as operators answer promptly [51].

One of the additional goals of implementing hotlines and call centers was to provide counseling services related to Covid-19. During the first wave of the pandemic, most countries experienced a rapid decrease in in-person medical consultations and an increase in remote consultations for primary and secondary care, with the majority of counseling sessions being conducted over the phone [13]. Neshnash et al. found that during the Covid-19 pandemic, the use of telephone counseling in primary healthcare has been timely and effective, with more than half of primary care physicians being satisfied or very satisfied with the telephone consultations [86]. In fact, remote counseling improves access to primary care and enables general practitioners to see patients who require an in-person appointment more quickly [86]. Similarly, Green et al.‘s study revealed that telephone counseling in the UK has nearly tripled from February 2020 to August 2021 [87].

One of the other services provided by hotlines and call centers was the Covid-19 triage. Ray et al. acknowledged that this type of triage can help to reduce unnecessary hospital visits and, as a result, minimize unnecessary face-to-face interactions. More importantly, this method guaranteed optimal utilization of test kits due to the shortage of testing kits during the initial months of the pandemic [88]. The main challenges mentioned in the studies were limitations in physical examination of patients, unavailability of hotlines/helplines/call centers, diverse clinical backgrounds of operators and counselors, and the absence of a previous protocol and a successful model for implementing hotlines. Similarly, other studies have highlighted the difficulty of diagnosing patients without physical examination and visual data [89–91].

The lessons learned indicated that in order to implement successful hotlines/helplines, certain factors such as ease of use and free access, sufficient human resources, staff training, adequate financial and physical resources, video tools and platforms for objective clinical assessment of the callers, having a translator, and receiving feedback from the stakeholders should be considered. Hasani et al. demonstrated that staff training is essential for high-quality and sustainable telephone counseling services in primary health care (PHC). Additionally, availability of translators for some patients is necessary [92]. Similarly, the lessons learned in Neshnash’s study showed that in order to achieve safe and effective remote consultation, healthcare systems must provide primary healthcare providers with adequate training and implement a national digital health framework for ensuring continuity of care [86]. Other researchers showed that a lack of sufficient training or a lack qualification for helpline operators may adversely impact the mental health of callers. Therefore, in order to improve the quality and performance of helplines, the development of short-term training courses for volunteers along with assessing their knowledge and skills through the output of exams, is necessary. Additionally, customer satisfaction feedback should be collected and reported periodically [93]. Overall, it seems that hotlines/helplines/call centers have been identified as an essential part of strategic response to crises. Findings demonstrated that many countries have used hotlines to support, and provide reliable information to the public.

### Research limitations

In this systematic review, only studies published in English were included. Therefore, research articles published in languages other than English were excluded due to the time and resource constraints. In addition, despite making efforts to get access to the full texts of articles and contacting the authors, the full texts of a number of studies were not available and we had to exclude them from the current study.

## Conclusion

The aim of this study was to investigate the characteristics, challenges and lessons learned of implementing Covid-19 hotlines/helplines/call centers during the pandemic. The results indicated that most hot lines/helplines/call centers were launched simultaneously with the official announcement of the coronavirus pandemic in March 2020 and were mostly accessible to the public seven days a week. The majority of callers included the public, hospital staff, healthcare providers, patients, families of patients, and elderly individuals. The services provided by call centers and phone lines primarily focused on providing psychological support, providing information to the individuals, answering their questions, offering counseling services, and triage. However, the implementation of these services faced challenges such as service unavailability, outdated information, limitations in physical examination of the callers, budget constraints, and the lack of standardized protocols. Factors such as providing ease of use and free access, allocating sufficient human resources, staff training, adequate financial and physical resource allocation, using video tools and platforms for objective examination of the callers, having a translator, and receiving feedback from stakeholders were reported as lessons learned in the relevant studies. It appears that healthcare managers and policy makers can utilize the findings of this study for successful implementation of hotlines/helplines/call centers in future crises. Therefore, developing strategies for future demands and conducting further research on the performance of current hotlines/helplines/call centers and experiences of the callers/operators are recommended.

### Electronic supplementary material

Below is the link to the electronic supplementary material.

Supplementary Material 1

## Data Availability

The data generated and analyzed during this systematic review are available from the corresponding author on reasonable request.

## Abbreviations

SARS: Severe Acute Respiratory Syndrome
MERS: Middle East Respiratory Syndrome
WHO: World Health Organization
MeSH: Medical Subjects Headings
PRISMA: Preferred Reporting Items for Systematic Reviews and Meta-analyses
AXIS: Appraisal tool for Cross-Sectional Studies
MMAT: Mixed Methods Appraisal Tool
CASP: Critical Appraisal Skills Programme
PSSHMS: Psychosocial Support and Mental Health Services
PSU: Psychological Support Unit
MHCH: Mental Health Crisis Helpline
UASD: University of Santo Domingo
LAMIC: Lower and Middle-Income Countries
PHC: Primary Health Care
