# Supplementary material for: Covid-19 hotlines, helplines and call centers: a systematic review of characteristics, challenges and lessons learned
Source: BMC Public Health. 2024 Apr 28;24:1191. doi: 10.1186/s12889-024-18702-8 (PMC11056073; doi:10.1186/s12889-024-18702-8)
Supplement: Supplementary file 1 — Supplementary Material 1 [file 12889_2024_18702_MOESM1_ESM.docx]

**Appendix I. Search strategy**

| Database | Search strategy |
| --- | --- |
| PubMed | **("COVID-19"[Title/Abstract] OR Corona[Title/Abstract] OR "Severe Acute Respiratory Syndrome"[Title/Abstract] OR "SARS-CoV-2"[Title/Abstract] OR "2019-nCoV"[Title/Abstract] OR Coronavirus[Title/Abstract] OR Covid[Title/Abstract]) AND (hotline[Title/Abstract] OR "call Center"[Title/Abstract] OR helpline[Title/Abstract] OR "Crisis line"[Title/Abstract] OR "Emergency line"[Title/Abstract])** |
| Web of Science | (TI=((("COVID-19" OR "Corona" OR "Severe Acute Respiratory Syndrome" OR "SARS-CoV-2" OR "2019-nCoV" OR "Coronavirus" OR "Covid") AND ("hotline" OR "call Center" OR "helpline" OR "Crisis line" OR "Emergency line")))) OR AB=((("COVID-19" OR "Corona" OR "Severe Acute Respiratory Syndrome" OR "SARS-CoV-2" OR "2019-nCoV" OR "Coronavirus" OR "Covid") AND ("hotline" OR "call Center" OR "helpline" OR "Crisis line" OR "Emergency line"))) |
| Scopus | **TITLE-ABS ( ( ( "COVID-19" OR "Corona" OR "Severe Acute Respiratory Syndrome" OR "SARS-CoV-2" OR "2019-nCoV" OR "Coronavirus" OR "Covid" ) AND ( "hotline" OR "call Center" OR "helpline" OR "Crisis line" OR "Emergency line" ) ) )** |
| Cochrane Library | ("COVID-19" OR "Corona" OR "Severe Acute Respiratory Syndrome" OR "SARS-CoV-2" OR "2019-nCoV" OR "Coronavirus" OR "Covid") AND ("hotline" OR "call Center" OR "helpline" OR "Crisis line" OR "Emergency line") in Record Title OR ("COVID-19" OR "Corona" OR "Severe Acute Respiratory Syndrome" OR "SARS-CoV-2" OR "2019-nCoV" OR "Coronavirus" OR "Covid") AND ("hotline" OR "call Center" OR "helpline" OR "Crisis line" OR "Emergency line") in Abstract - in Cochrane Reviews, Trials (Word variations have been searched) |
| IEEE xplore | ("Document Title":"COVID-19" OR "Document Title":"Corona" OR "Document Title":"Severe Acute Respiratory Syndrome" OR "Document Title":"SARS-CoV-2" OR "Document Title":"2019-nCoV" OR "Document Title":"Coronavirus" OR "Document Title":"Covid") AND ("Document Title":"hotline" OR "Document Title":"call Center" OR "Document Title":"helpline" OR "Document Title":"Crisis line" OR "Document Title":"Emergency line") OR ("Abstract":"COVID-19" OR "Abstract":"Corona" OR "Abstract":"Severe Acute Respiratory Syndrome" OR "Abstract":"SARS-CoV-2" OR "Abstract":"2019-nCoV" OR "Abstract":"Coronavirus" OR "Abstract":"Covid") AND ("Abstract":"hotline" OR "Abstract":"call Center" OR "Abstract":"helpline" OR "Abstract":"Crisis line" OR "Abstract":"Emergency line") |
| ProQuest | title((("COVID-19" OR "Corona" OR "Severe Acute Respiratory Syndrome" OR "SARS-CoV-2" OR "2019-nCoV" OR "Coronavirus" OR "Covid") AND ("hotline" OR "call Center" OR "helpline" OR "Crisis line" OR "Emergency line"))) OR abstract((("COVID-19" OR "Corona" OR "Severe Acute Respiratory Syndrome" OR "SARS-CoV-2" OR "2019-nCoV" OR "Coronavirus" OR "Covid") AND ("hotline" OR "call Center" OR "helpline" OR "Crisis line" OR "Emergency line"))) |
| Google Scholar | allintitle: "Covid-19" "hotline" OR "helpline" OR "call center" |

**Appendix II- Quality and risk of bias assessment**

**The Appraisal tool for Cross-Sectional Studies for quantitative studies (Yes, No, Do not know)**

| Criteria | Bric and Raile [60] | Carson et al. [53] | Kumar et al. [62] | Margolius et al [54] | Pelicon et al. [64] | Ravindran et al. [37] | Shao et al. [44] | Cher et al. [55] | Jang et al. [20] | Egić [67] | Khan et al. [39] | Monreal-Bartolomé et al. [68] | Nina-Mollinedo et al. [22] | Singh et al. [52] | Sosa Lovera et al. [59] | Abdelbaky et al. [70] | Alabdulla et al. [48] | Arafa et al. [49] | Gussin et al. [57] | Ibrahim et al. [42] | Lai et al. [47] | Tansa et al. [43] | Thangarasu et al. [41] |
| --- | --- | --- | --- | --- | --- | --- | --- | --- | --- | --- | --- | --- | --- | --- | --- | --- | --- | --- | --- | --- | --- | --- | --- |
| 1. Were the aims/objectives of the study clear? | Yes | Yes | Yes | Yes | Yes | Yes | No | Yes | Yes | Yes | Yes | Yes | Yes | Yes | Yes | Yes | Yes | Yes | Yes | Yes | Yes | Yes | Yes |
| 2. Was the study design appropriate for the stated aim(s)? | Yes | Yes | Yes | Yes | Yes | Yes | Yes | Yes | Yes | Yes | Yes | Yes | Yes | Yes | Yes | Yes | Yes | Yes | Yes | Yes | Yes | Yes | Yes |
| 3. Was the sample size justified? | Yes | No | Yes | Yes | Yes | Do not know | Do not know | Yes | Yes | Yes | No | Yes | Yes | Yes | Yes | Yes | Yes | Yes | Yes | Yes | Yes | Yes | Yes |
| 4. Was the target/reference population clearly defined? (Is it clear who the research was about?) | Yes | Yes | Yes | Yes | Yes | Yes | Yes | Yes | Yes | Yes | Yes | Yes | Yes | Yes | Yes | Yes | Yes | Yes | Yes | Yes | Yes | Yes | Yes |
| 5. Was the sample frame taken from an appropriate population base so that it closely represented the target/reference population under investigation? | Yes | Yes | Yes | Yes | Yes | Yes | Yes | Yes | Yes | Yes | Yes | Yes | Yes | Yes | Yes | Yes | Yes | Yes | Yes | Yes | Yes | Yes | Yes |
| 6. Was the selection process likely to select subjects/participants that were representative of the target/reference population under investigation? | Yes | Yes | Yes | Yes | Yes | Do not know | Yes | Yes | Yes | Yes | No | Yes | Yes | Yes | Yes | Yes | Yes | Yes | Yes | Yes | Yes | Yes | Yes |
| 7. Were measures undertaken to address and categorise non-responders? | Do not know | Do not know | Do not know | Yes | Do not know | No | Do not know | Do not know | No | No | No | No | No | Do not know | No | Do not know | Do not know | Do not know | Do not know | Yes | Do not know | Do not know | Do not know |
| 8. Were the risk factor and outcome variables measured appropriate to the aims of the study? | Do not know | Do not know | Do not know | Yes | Do not know | No | Do not know | Do not know | Yes | Yes | Yes | Yes | Yes | Yes | Yes | Yes | Yes | Yes | Yes | Yes | Yes | Yes | Yes |
| 9. Were the risk factor and outcome variables measured correctly using instruments/ measurements that had been trialled, piloted or published previously? | Do not know | Do not know | Do not know | Yes | Do not know | No | Do not know | Do not know | Yes | Yes | Do not know | Yes | Yes | Yes | Do not know | Yes | Yes | Yes | Yes | Yes | Yes | Yes | Yes |
| 10. Is it clear what was used to determined statistical significance and/or precision estimates? (eg, p values, CIs) | Yes | Yes | Yes | Yes | Yes | No | Yes | Yes | Yes | Yes | Yes | Yes | Yes | Yes | Yes | Yes | Yes | Yes | Yes | Yes | Yes | Yes | Yes |
| 11. Were the methods (including statistical methods) sufficiently described to enable them to be repeated? | Yes | Yes | No | Yes | Yes | No | No | Yes | Yes | No | No | Yes | Yes | Yes | No | Yes | Yes | Yes | Yes | No | Yes | No | Yes |
| 12. Were the basic data adequately described? | Yes | Yes | Yes | Yes | Yes | Yes | Yes | Yes | Yes | Yes | Yes | Yes | Yes | Yes | Yes | Yes | Yes | Yes | Yes | Yes | Yes | Yes | Yes |
| 13. Does the response rate raise concerns about non-response bias? | Do not know | Do not know | Yes | Yes | Do not know | Do not know | Yes | Yes | Yes | Yes | Do not know | Yes | Yes | Do not know | No | Yes | Do not know | Yes | Do not know | Do not know | Do not know | Do not know | Do not know |
| 14. If appropriate, was information about non-responders described? | Do not know | No | Do not know | No | No | No | No | Do not know | No | No | No | No | Yes | No | Yes | No | Yes | Yes | Do not know | Yes | Do not know | Do not know | Do not know |
| 15. Were the results internally consistent? | Yes | Yes | Yes | Yes | Yes | Yes | Yes | Yes | Yes | Yes | Yes | Yes | Yes | Yes | Yes | Yes | Yes | Yes | Yes | Yes | Yes | Yes | Yes |
| 16. Were the results for the analyses described in the methods, presented? | Yes | Yes | Yes | Yes | Yes | Yes | Yes | Yes | Yes | Yes | Yes | Yes | Yes | Yes | Yes | Yes | Yes | Yes | Yes | Yes | Yes | Yes | Yes |

**Quality assessment using MMAT for mixed methods studies (Yes, No, Can’t tell)**

| Criteria | Geoffroy et al. [61] | Matthewson et al. [63] | Abdullah et al. [29] | Cheng et al. [31] | Du et al. [30] | Hazarika et al. [38] | Iqbal et al. [50] | Meaden et al. [56] | Wahl et al. [65] | Zabrzygraj and Świtaj [16] | Alfatih and Rachmawati [51] | Ouyang et al. [45] | Peng et al. [46] | Bates et al. [66] | Kok et al. [71] | Munyikwa et al. [58] | Sasidharan et al. [40] |
| --- | --- | --- | --- | --- | --- | --- | --- | --- | --- | --- | --- | --- | --- | --- | --- | --- | --- |
| 1. Is there an adequate rationale for using a mixed methods design to address the research question? | Yes | Yes | Yes | Yes | Yes | Yes | Yes | Yes | Yes | Yes | Yes | Yes | Yes | Yes | Yes | Yes | Yes |
| 2. Are the different components of the study effectively integrated to answer the research question? | Yes | Yes | Yes | Yes | Yes | Yes | Yes | Yes | Yes | Yes | Yes | Yes | Yes | Yes | Yes | Yes | Yes |
| 3. Are the outputs of the integration of qualitative and quantitative components adequately interpreted? | Yes | Yes | Yes | No | Yes | Yes | Yes | Yes | Yes | Yes | Yes | Yes | Yes | Yes | Yes | Yes | Yes |
| 4. Are divergences and inconsistencies between quantitative and qualitative results adequately addressed? | Yes | Yes | Yes | Yes | Yes | Yes | Yes | Yes | Yes | Yes | Yes | Yes | Yes | Yes | Yes | Yes | Yes |
| 5. Do the different components of the study adhere to the quality criteria of each tradition of the methods involved? | Yes | Yes | Yes | Yes | Yes | Yes | Yes | Yes | Yes | Yes | Yes | Yes | Yes | Yes | Yes | Yes | Yes |

**The Critical Appraisal Skills Programme (CASP) Checklist for qualitative research (Yes, No, Can’t Tell)**

| Criteria | Joshi et al. [4] | Kristal et al. [17] | Abdelghaffar et al. [69] |
| --- | --- | --- | --- |
| 1. Was there a clear statement of the aims of the research? | Yes | No | Yes |
| 2. Is a qualitative methodology appropriate? Is it worth continuing? | Yes | Yes | Yes |
| 3. Was the research design appropriate to address the aims of the research? | Yes | Yes | Yes |
| 4. Was the recruitment strategy appropriate to the aims of the research? | Yes | Yes | Yes |
| 5. Was the data collected in a way that addressed the research issue? | Yes | Yes | Yes |
| 6. Has the relationship between researcher and participants been adequately considered? | Yes | Can’t Tell | Can’t Tell |
| 7. Have ethical issues been taken into consideration? | Yes | Can’t Tell | Can’t Tell |
| 8. Was the data analysis sufficiently rigorous? | Yes | Yes | Can’t Tell |
| 9. Is there a clear statement of findings? | Yes | Yes | No |
| 10. How valuable is the research? | Yes | Yes | Yes |
